# Kinetic trapping of a cobalt(ii) metallocage using a carbazole-containing expanded carbaporphyrinoid ligand†

**DOI:** 10.1039/d1sc06514a

**Published:** 2021-12-20

**Authors:** Weinan Zhou, Tridib Sarma, Yonghuan Su, Chuanhu Lei, Jonathan L. Sessler

**Affiliations:** School of Materials Science and Engineering, Shanghai University Shanghai 200444 China; Center for Supramolecular Chemistry and Catalysis and Department of Chemistry, College of Science, Shanghai University Shanghai 200444 China chlei@shu.edu.cn; Department of Chemistry, Cotton University Guwahati 781001 Assam India; Department of Chemistry, The University of Texas at Austin 105 East 24th Street, Stop A5300 Austin Texas 78712-1224 USA sessler@cm.utexas.edu

## Abstract

The *meso*-unsubstituted expanded porphyrinoid 3, incorporating two carbazole moieties, acts as an effective ligand for Co(ii) and permits the isolation and X-ray diffraction-based characterization of a 6 : 3 metal-to-ligand metallocage complex that converts spontaneously to the constituent 2 : 1 metal-to-ligand metalloring species in chloroform solution. The discrete metalloring is formed directly when the Co(ii) complex is crystallized from supersaturated solutions, whereas crystallization from more dilute solutions favors the metallocage. Studies with two other test cations, Pd(ii) and Zn(ii), revealed exclusive formation of the monomeric metalloring complexes with no evidence of higher order species being formed. Structural, electrochemical and UV-vis-NIR absorption spectral studies provide support for the conclusion that the Pd(ii) complex is less distorted and more effectively conjugated than its Co(ii) and Zn(ii) congeners, an inference further supported by TD-DFT calculations. The findings reported here underscore how expanded porphyrins can support coordination modes, including bimetallic complexes and self-assembled cage structures, that are not necessarily easy to access using more traditional ligand systems.

## Abstract 2

Carbazole containing expanded carbaporphyrinoid ligand supports the formation of 2 : 1 metal-to-ligand complexes with Pd, Co, and Zn. Solid-state studies also revealed formation of a 6 : 3 metal-to-ligand metallocage in the case of Co complexation.

## Graphical abstract

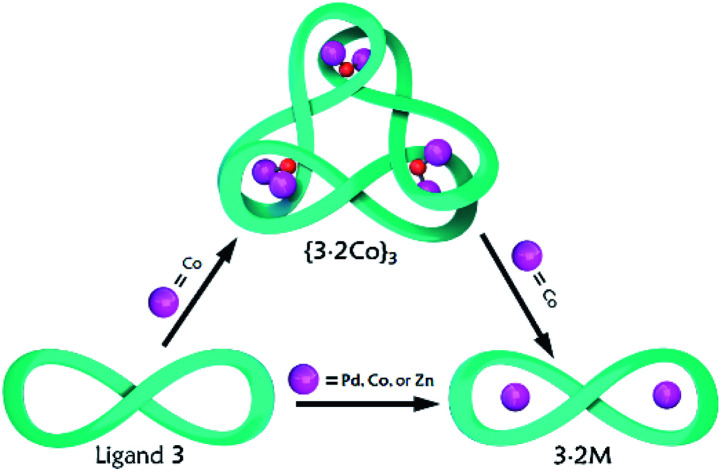

## Introduction

**Scheme 1:**
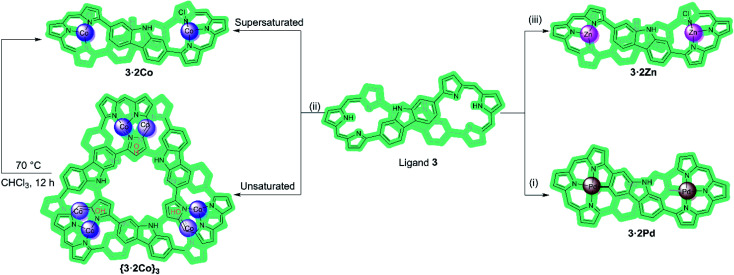
Metalation of ligand 3 with Pa(ii), Co(ii), and Zn(ii) ions. Reagents and conditions: (i), (ii), and (iii) M(OAc)_2_, NaOAc, CHCl_3_/MeOH, RT. β-Pyrrolic alkyl groups are omitted for clarity.

Over the past two decades considerable efforts have been devoted to exploring the metal cation coordination chemistry of expanded porphyrins.^1–3^ This has led to advances that are not easily recapitulated in the case of other ligand systems. For instance, metalation of expanded porphyrins has been used to trigger changes in electronic structure, including the conversion between nonaromatic and aromatic forms.^4–6^ In addition, expanded porphyrins have been used for metal ion recognition and sensing;^7–11^ they have also been incorporated into stimuli responsive molecular machines.^12^ Other metallated expanded porphyrins have been investigated as catalysts for organic synthesis^13,14^ and as functional photoacoustic imaging agents.^15^ In 2014, Lash and coworkers described a so-called *adj*-dicarbaporphyrin that stabilises an unusual tris-palladium sandwich complex.^16^ However, as a general rule, and in contrast to what is true for porphyrins *per se*,^17–19^ the use of expanded porphyrins to self-assemble multi-metallated arrays is all but unexplored. Moreover, to our knowledge no expanded porphyrin has been used to trap a structurally characterized metallocage as an inherently unstable kinetic product. Here, we report that Co(ii) complexation of a *meso*-unsubstituted expanded porphyrinoid incorporating two carbazole moieties (3)^20^ can, under appropriately chosen conditions, produce a 6 : 3 metal-to-ligand hexa-Co(ii) metallocage ({3·2Co}_3_) comprising three bis-metallated expanded porphyrin subunits. This metallocage converts spontaneously in CHCl_3_ solution to the corresponding single component bis-Co(ii) complex (3·2Co), a thermodynamically favored product that is readily obtained by independent synthesis. Discrete bis-metallated metalloring species (3·2M) are obtained exclusively in our hands when the Co(ii) source, Co(OAc)_2_, is replaced by M(OAc)_2_, where M = Pd(ii) or Zn(ii) (Scheme 1). The present study demonstrates how the judicious interplay between expanded porphyrin ligand design and coordination chemistry can be used to access metal-containing ensembles that lie off equilibrium with regard to their more thermodynamically stable monomeric forms.

The present study relies on the use of a bis-carbazole expanded porphyrin (ligand 3). Carbazole-containing expanded porphyrins may be viewed as being a class of expanded carbaporphyrins,^21,22^ systems with one or more carbon donors incorporated into the central core.^23–29^ These unique macrocycles exhibit a diverse range of properties, including unusual aromaticity and chirality features,^30–32^ as well as serving as potential ligands.^33–36^ Among the various expanded carbaporphyrinoids reported to date, those with two embedded polyaromatic hydrocarbons or related heterocycles (PAHs) are still relatively rare and their metalation chemistry has not been extensively explored.^37,38^ Recently, we reported the synthesis of the *meso*-free expanded carbaporphyrinoid 3. In its as-prepared free-base form, 3 proved to be a flexible macrocycle that adopts figure-of-eight conformations that provide two tripyrrane-like pockets. We considered it likely that this system would prove useful as a ligand. What was less apparent was whether it would support chemistry that extended beyond the first coordination sphere. The present study was undertaken in an effort to test this possibility.

## Results and discussion

Macrocycle 3 was prepared as detailed previously.^20^ Initially, we examined Pd(ii) metalation (Scheme 1). Insertion of Pd(ii) ions into ligand 3 was performed by treating with 10 molar equiv. of both palladium acetate and sodium acetate in CHCl_3_/MeOH (4 : 1) at room temperature for 8 h. After purification over neutral alumina followed by recrystallization from CH_2_Cl_2_/MeOH, the bis-Pd(ii) complex (3·2Pd) was isolated in 68% yield. A MALDI-TOF mass spectrometric analysis revealed mass peaks at *m*/*z* = 1314.3255 ([M]^+^); calcd for C_76_H_76_N_8_Pd_2_: 1314.4267 (Fig. S10†).

**Fig. 1:**
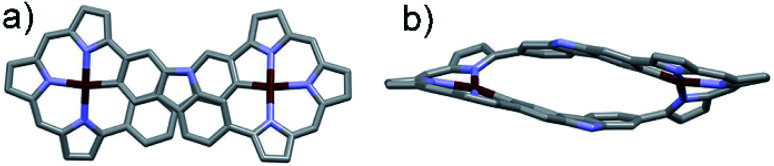
Single crystal X-ray structures of 3·2Pd. (a) Top and (b) side views. Atom color key: carbon (light grey), nitrogen (light purple), palladium (maroon). Hydrogen atoms, β-pyrrolic alkyl groups and solvent molecules have been omitted for clarity.

Diffraction grade single crystals of 3·2Pd were obtained *via* the slow diffusion of methanol into a dichloromethane solution of the complex. An X-ray diffraction analysis revealed a solid-state structure possessing *C*_i_ molecular symmetry and a twisted figure-of-eight shape analogous to the free ligand 3 (Fig. 1). The two Pd(ii) ions are each bound to three pyrrolic nitrogen atoms and one carbazole carbon atom. This leads to a slightly distorted square-planar NNNC coordination geometry with a *τ*_4_ (ref. 39) value = 0.17 reminiscent of what was seen in a previously reported core modified octaphyrin Pd(ii) complex.^29^ Two independent structures are seen in the asymmetric unit, but they do not differ substantially (Fig. S15 and S16†). The distances between the palladium ions seen in these structures are 10.129 Å and 10.161 Å, respectively, whereas the Pd–N bond lengths vary from 1.959 to 2.095 Å and from 1.947 to 2.091 Å in these two structures, respectively. Likewise, Pd–C bond lengths of 2.037 and 2.041 Å, and 2.035 and 2.048 Å are found.

**Fig. 2:**
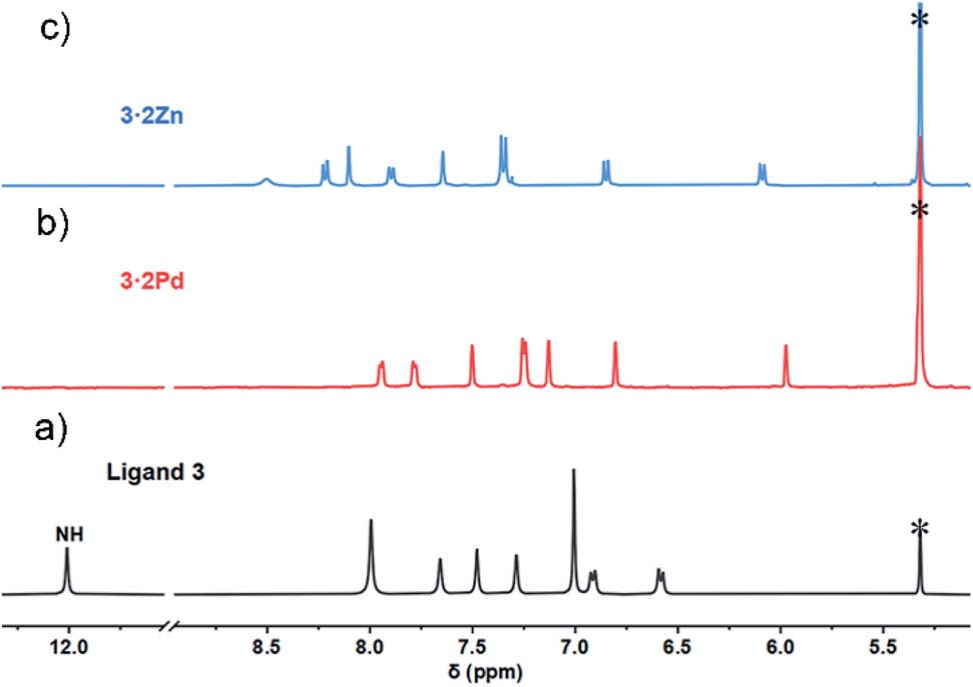
Comparative ^1^H NMR spectra (aromatic region) of ligand 3 and metal complexes. (a) Ligand 3 at −60 °C in CD_2_Cl_2_; (b) 3·2Pd at 25 °C in CD_2_Cl_2_; (c) 3·2Zn at −60 °C in CD_2_Cl_2_. Residual solvents were marked with asterisks.

In contrast to what was seen for ligand 3, the ^1^H NMR spectrum of 3·2Pd recorded in CD_2_Cl_2_ is characterized by sharp signals at room temperature. Presumably, this reflects a system that is conformally rigid as the result of NNNC–Pd(ii) coordination (Fig. S1†). Specific peak assignments were made on the basis of COSY and NOESY experiments (Fig. S4 and S5†). The chemical shifts of 3·2Pd are reminiscent of those seen for ligand 3 (Fig. 2). Of note are the absence of both pyrrolic NH signals, and the presence of two doublets and four singlets ascribed to the carbazole protons in the range of 5.97–7.95 ppm that further corroborated coordination occurs consistent with solid state structure. Bifurcated peaks corresponding to the four *meso* CH protons are also seen at 6.80 and 7.13 ppm that might reflect the *C*_i_ molecular symmetry of 3·2Pd and the chemical inequivalence of the *meso* CH protons.

**Fig. 3:**
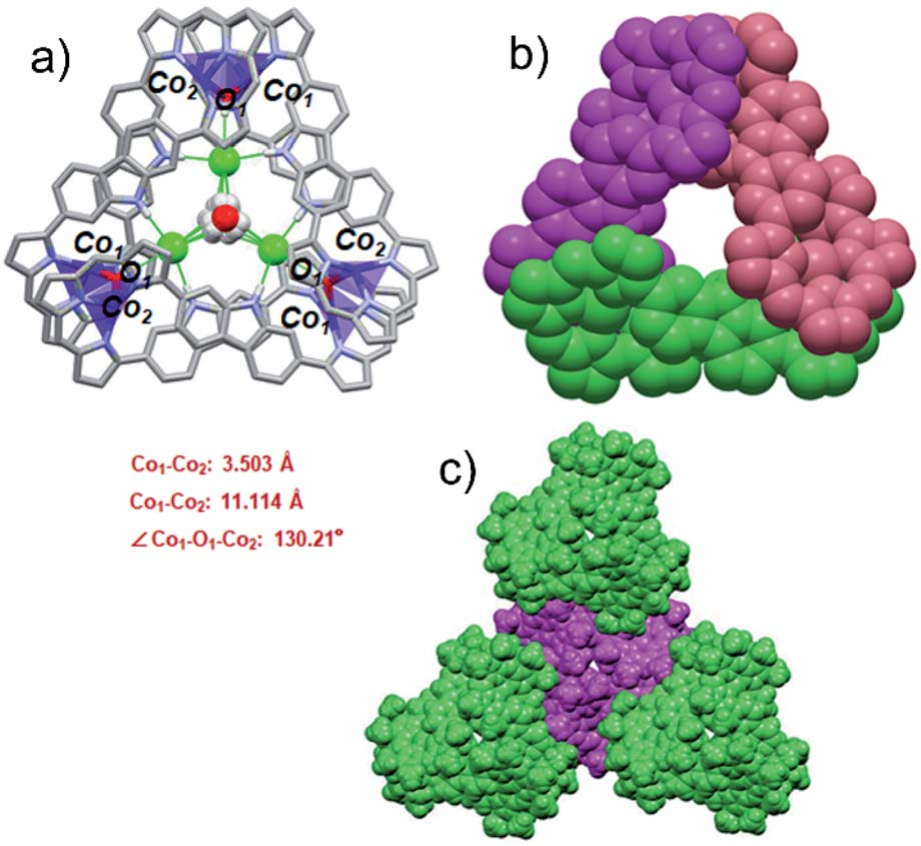
Single crystal X-ray structures of {3·2Co}_3_. (a) Top view of {3·2Co}_3_. Atom color key: hydrogen (white), carbon (light gray), nitrogen (light purple), cobalt (violet), chloride (green). Hydrogen atoms that are not involved in intermolecular interactions, β-pyrrolic alkyl groups and solvent molecules are omitted for clarity. (b) Top view in space-filling form showing individual 3·2Co subunits (each colored differently for clarity). (c) Top view of molecular packing arrangement showing individual {3·2Co}_3_ cages and the overall spiral arrangement (individual cages are colored differently for clarity).

Co(ii) metalation of ligand 3 was performed following the procedure used to effect Pd(ii) insertion. Diffraction grade single crystals were then obtained by allowing *n*-hexane to diffuse slowly into a chloroform solution of 3·2Co. Structural analysis (Fig. 3a) revealed that under these conditions three units of [3·2Co–2Cl] and three hydroxy ligands trimerize to give a triangular-shaped metallocage that includes two water molecules and three chloride ions within its central cavity. This unique structure in the solid state may make this cage of interest in the context of chloride anion capture.^40^

In the cage structure, referred to as {3·2Co}_3_, the three constituent 3·2Co monomers retain their figure-of-eight shape and are interconnected in a staggered manner. This results in overall *C*_3_ symmetry (Fig. 3b). In each monomer, the two Co ions are each bound to three tripyrrin-derived nitrogen atoms and share an axial hydroxy ligand with another monomer. Three axially coordinated oxygen atoms define a regular triangle defined by an O–O distance of 10.516 Å. The Co–O band lengths are 1.93(1) Å. Two water molecules are located above and below the mean plane (with respect to the three oxygen atoms) at distances of 1.617 Å and 1.719 Å, respectively. The two water molecules share three common chloride ions connected through O–H⋯Cl hydrogen bonds (av. OH⋯Cl: 2.581 Å). In addition, three chloride ions are directly bound to the carbazole NH protons (av. NH⋯Cl: 2.487 Å) and to axial OH moieties (av. OH⋯Cl: 2.339 Å) *via* an intermolecular hydrogen-bonding network. It is likely that both the chloride anions and water molecules help stabilize the cage structure. As shown in Fig. 3c, in the solid state each molecule cage unit ({3·2Co}_3_) is linked to three nearest neighbours *via* what are inferred to be weak intermolecular van der Waals interactions.

We next modified the crystallization conditions. Specifically, Co(OAc)_2_ (45 mg), NaOAc (15 mg), and ligand 3 (20 mg) in a mixture of CHCl_3_/MeOH (4 : 1; 20 mL) were allowed to react for 8 h at room temperature. After workup and purification, metallic rhombic-like crystals of 3·2Co were obtained by diffusion of *n*-hexane (liquid) into a supersaturated dichloromethane solution of the complex over the course of 3–5 days in a sealed vial at room temperature (Fig. S24a†). In contrast, when the concentration of the complex in the chloroform solution was kept low and slow vapor diffusion of hexane into a chloroform solution of the complex at room temperature was used to promote crystallization, brick-like crystal of {3·2Co}_3_ analogous to those generated originally were obtained after about three weeks (Fig. S24b†).

**Fig. 4:**
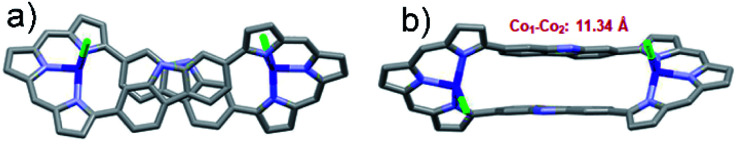
Single crystal X-ray structures of 3·2Co. (a) Top and (b) side views. Atom color key: carbon (light grey), nitrogen (light purple), cobalt (violet), chloride (green). Hydrogen atoms, β-pyrrolic alkyl groups and solvent molecules have been omitted for clarity.

Crystallographic analysis (Fig. 4) of the crystals considered to consist of 3·2Co revealed a *C*_2_ symmetric figure-of-eight structure similar to that for the free ligand 3^20^ and the 3·2Pd complex described above. Both Co ions are coordinated to three tripyrrane-like nitrogen atoms and an axial chloride. This results in a distorted tetrahedral coordination geometry with a *τ*_4_ value of 0.74. The distance between the Co centres is approximately 11.34 Å. The Co–N bond lengths vary from 1.967 to 2.027 Å, whereas the Co–Cl bond lengths range from 2.291 to 2.324 Å. The ^1^H NMR spectrum of 3·2Co revealed peaks at extremely low field (*ca.* 50 ppm) reflecting the paramagnetic effect of the coordinated Co(ii) ions (Fig. S6†). Variable temperature magnetic susceptibility analyses further proved consistent with the two Co ions being in the +2, *S* = 3/2 high oxidation/spin state. A single-core model that did not account for possible intramolecular magnetic exchange interactions was used to fit the magnetic properties of 3·2Co; this gave values for *ZJ* and *g* of −0.033 and 2.57 cm^−1^, respectively (Fig. S35†). Unfortunately, the magnetic parameters of {3·2Co}_3_ could not be determined due to its instability; *vide infra*.

**Fig. 5:**
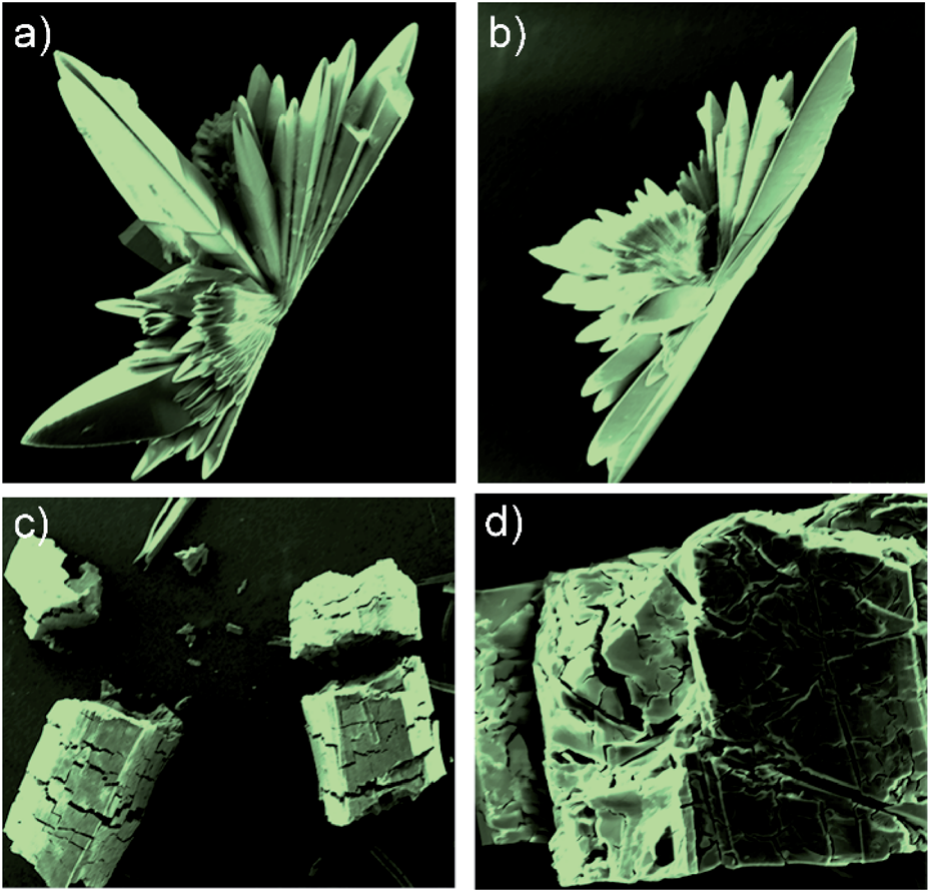
SEM images of the crystalline entities: (a) and (b) 3·2Co; (c) and (d) {3·2Co}_3_.

The two different crystalline species {3·2Co}_3_ and 3·2Co were analysed by scanning electron microscopy (SEM) (Fig. 5, S25 and S26†). These analyses revealed that crystals of {3·2Co}_3_ were prone to collapse or fracture even in the solid state. This provided a preliminary indication that this form might not be thermodynamically stable relative to the monomeric bis-Co(ii) form, 3·2Co.

Consistent with the above inference, time-dependent UV-vis spectroscopic analyses revealed that the metallocage {3·2Co}_3_ decomposed gradually in CHCl_3_ solution at room temperature even when starting from pure crystals ({3·2Co}_3_) (Fig. S29†). We then confirmed that the metallocage {3·2Co}_3_ is transformed into the corresponding metalloring complex 3·2Co in CHCl_3_ solution at room temperature (Fig. S30†). Subjecting {3·2Co}_3_ to reflux in CHCl_3_ solution accelerated this transformation (Fig. S14 and S31†). HR-FT-ICR MS analyses revealed that the 2 : 1 complex produced as the result of this transformation bears a coordinated OH group on each Co(ii) centre (Fig. S13†). We thus conclude that {3·2Co}_3_ lies off-equilibrium with respect to its constituent 3·2Co subunits. The relatively facile interconversion is ascribed to the lability of the coordination bonds in {3·2Co}_3_, which in other instances can allow for stimulus-induced structural rearrangements to give species of differing sizes and shapes.^41^ On the basis of the Gibbs free energy calculation, the corresponding Δ*G* value for going from the {3·2Co}_3_ cage to the 3·2Co macrocycle is −14.601 kcal mol^−1^, supporting the conclusion that the macrocycle is the thermodynamically favoured product (Fig. S42†).

In an effort to probe further the coordination chemistry of ligand 3, we explored its ability to stabilize complex(es) with Zn(ii), a cation prone to form labile ligand–metal bonds. Metalation of 3 with zinc acetate, afforded the corresponding bis-metal complex, 3·2Zn, with no evidence of higher order species being detected *via* MALDI-TOF MS analysis (Fig. S12†). Single crystals of 3·2Zn were obtained through slow diffusion of methanol into a chloroform solution. The structure of 3·2Zn was confirmed by X-ray diffraction analysis and proved similar to that of 3·2Co (Scheme 1 and Fig. S19†).

The ^1^H NMR spectrum of 3·2Zn recorded in CD_2_Cl_2_ solution is characterized by a rather broad signal pattern in the aromatic region (Fig. S7†). This finding is consistent with a system that is conformationally flexible on the NMR time scale at ambient temperature. At −60 °C the ^1^H NMR spectrum becomes sharp with seven distinct set of protons, ascribed to the carbazole subunit, being observed in the range of 6.0–8.5 ppm region (Fig. 2). Bifurcated signal corresponding to the four *meso* CH protons are seen at 7.35 ppm. All signals were further assigned by COSY and NOESY experiments (Fig. S8 and S9†). The ^1^H NMR spectral analyses thus provide support for the conclusion, also drawn from the X-ray diffraction study above, that the basic structural features of ligand 3 are preserved upon Zn ion complexation.

**Fig. 6:**
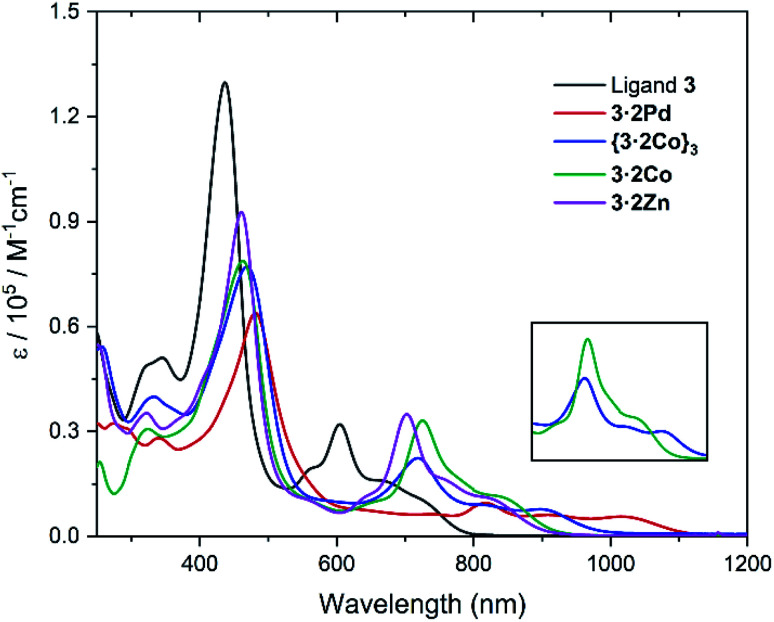
UV-vis-NIR absorption spectra of ligand 3, 3·2Pd, {3·2Co}_3_, 3·2Co, and 3·2Zn in CHCl_3_ at room temperature. (inset) Comparative of {3·2Co}_3_ and 3·2Co expanded region at 600–1000 nm.

The absorption spectra of ligand 3 and its corresponding metal complexes 3·2Pd, {3·2Co}_3_, 3·2Co, and 3·2Zn were measured in chloroform at room temperature. As can be seen from an inspection of Fig. 6 and Table 1, relative to ligand 3, the UV-vis-NIR absorption spectra of the metal complexes exhibit distinct bathochromic shifts, particularly in the NIR region; this is thought to reflect metal ion insertion into the cavity that serves to strengthen the extent of conjugation within the macrocycle.^42^ The absorption features of the metallocage {3·2Co}_3_ and metalloring 3·2Co were qualitatively similar, although differences are seen in the absorption tails, which extend out to *ca.* 1010 nm and *ca.* 960 nm in the case of {3·2Co}_3_ and 3·2Co, respectively.

**Table 1:** Optical^a^ and electrochemical^b^ data for ligand 3 and complexes 3·2Pd, 3·2Co, and 3·2Zn

Compound	*λ* [nm] (*ε* [×10^5^ L mol^−1^ cm^−1^])	*E*_ox.3_	*E*_ox.2_	*E*_ox.1_	*E*_red.1_	*E*_red.2_	*E*_red.3_	Δ*E*_HL_^c^ [eV]	Δ*E*_HL_^d^ [eV]
3	436 (1.30), 605 (0.32)	—	0.43	0.33	−1.21	−1.50	—	1.54	1.90
3·2Pd	481 (0.64), 818 (0.10)	0.84	0.34	0.19	−1.07	−1.13	−1.71	1.26	1.67
3·2Co	463 (0.79), 725 (0.33)	1.13	0.58	0.49	−0.85	−1.15	−1.65	1.34	1.88
3·2Zn	461 (0.93), 702 (0.35)	1.17	0.58	0.49	−0.95	−1.18	—	1.44	1.89

a UV-vis-NIR absorption spectra were recorded in CHCl_3_ at room temperature.

b Cyclic voltammetry studies were conducted in CH_2_Cl_2_ containing 0.1 M *n*-Bu_4_NPF_6_ as the supporting electrolyte, Ag/Ag^+^ was used as the reference electrode, Pt wire was used as the counter electrode, and glassy carbon as the working electrode. Potentials were recorded *vs.* ferrocene/ferrocenium ion. Scan rates were 0.05 V s^−1^. These potentials were determined by differential pulse voltammetry.

c Electrochemical HOMO–LUMO energy gap [eV] = *E*_ox.1_ − *E*_red.1_.

d HOMO–LUMO energy gap obtained by TD-DFT calculations (B3LYP/6-311G(d,p)).

Particularly noteworthy are the low energy absorption maxima at 818 nm for Pd, 725 nm for Co, and 702 nm for Zn, respectively, which are approximately 100 nm to 200 nm bathochromically shifted as compared to the corresponding band in the case of ligand 3 (*λ*_max_ = 605 nm). Also of interest is that, compared with the Zn and Co complexes (3·2Co and 3·2Zn), the absorption of the Pd complex (3·2Pd) is broader and is characterized by a tail that extends to *ca.* 1100 nm. This is taken as an indication that the π-conjugation is relatively enhanced in the case of the bis-Pd complex, an inference consistent with the solid state analyses, which revealed less twisting between the carbazole units and the tripyrranes in the case of 3·2Pd (av. 20.8°, Fig. S16†) as compared to 3·2Co (av. 35.23°, Fig. S17†) and 3·2Zn (av. 36.09°, Fig. S20†).

The redox properties of ligand 3 and the corresponding bis-metal complexes were also examined *via* cyclic voltammetry (CV) and differential pulse voltammetry (DPV) (Fig. S32–35†). The resulting redox potentials and electrochemically-derived HOMO–LUMO gaps (Δ*E*_HL_) are summarized in Table 1. With respect to 3, two irreversible oxidation waves at 0.33 and 0.43 V, along with one reversible reduction wave at −1.21 V and one irreversible wave at −1.50 V, are seen. Upon metal insertion, reduction becomes more facile. The extent of this anodic shift is smallest in the case of 3·2Pd. The first oxidation wave for 3·2Pd (0.19 V) also remains more negative than those for 3·2Zn and 3·2Co (both 0.49 V). This results in the smallest HOMO–LUMO gap (1.26 eV), a finding consistent with the low energy absorption features seen for this complex. (Note: the lifetime of metallocage {3·2Co}_3_ in solution proved too short to allow its analysis by electrochemical means.)

Time-dependent density functional theory (TD-DFT) calculations revealed that the frontier molecular orbitals (FMOs) of 3·2Co and 3·2Zn are similar and that these complexes possess analogous HOMO–LUMO energy gaps (Fig. S43 and S44†). In the case of 3·2Pd (Fig. S43†), the HOMO energy level is elevated as compared to the 3·2Zn and 3·2Co complexes. This leads to a slight decrease in the band gap (Δ*E* = 1.67 eV) for 3·2Pd as compared to 3·2Zn and 3·2Co (Δ*E* = 1.88–1.90 eV), in good agreement with the electrochemical HOMO–LUMO gaps (Table 1) and the relatively larger red shift in the absorption spectral features seen for 3·2Pd. Across the board TD-DFT studies revealed a good correspondence between the observed and predicted spectral features (Fig. S46–48† and Tables S3–S5†).

## Conclusions

In summary, ligand 3 supports the formation of 2 : 1 metal-to-ligand complexes with Pd(ii), Co(ii), and Zn(ii) (3·2Pd, 3·2Co, and 3·2Zn, respectively). Single-crystal X-ray diffraction structural analyses revealed distorted square-planar geometries about the metal centres in 3·2Pd, but tetrahedral coordination modes in the case of 3·2Co and 3·2Zn. These differences translate into a lower level of ligand distortion and better conjugation in the case of the Pd(ii) complex, as inferred from spectroscopic and electrochemical analyses, as well as the TD-DFT calculations. In the case of Co(ii), crystallization conditions (*e.g.*, use of unsaturated solutions and antisolvent vapor diffusion) could be found that allowed for the kinetic trapping of a crystalline 6 : 3 metal-to-ligand metallocage ({3·2Co}_3_) containing three 3·2Co metallorings. This metallocage converts readily to the more thermodynamically favored 2 : 1 (3·2Co) metalloring form in CHCl_3_ solution. The present work thus highlights how expanded carbaporphyrinoids may be used to access structural forms, including self-assembled metal complexes, that are not necessarily accessible using simpler ligand systems.

## Data availability

Crystallographic data for {3·2Co}_3_, 3·2Zn, 3·2Co, and 3·2Pd have been deposited at the CCDC under accession numbers 2105486–2105488 and 2105812 respectively, and can be obtained from Home – The Cambridge Crystallographic Data Centre (CCDC). Cyclic voltammetry, magnetic susceptibility measurement, NMR and MS spectra, DFT calculation details and Cartesian coordinates of calculated complexes supporting this article have been uploaded as part of the ESI.†

## Author contributions

Conceptualization and supervision: JLS, TS, and CH; synthesis, characterization, NMR, spectroscopy, SEM, SQUID, CV and DPV studies: WN and YH; single crystal growing and data analysis: WN and CH; theoretical calculations: WN and XL; writing – original: WN; writing – review and editing: JLS, TS and CH. All authors proofread, commented on, and approved the final version of this manuscript.

## Conflicts of interest

There are no conflicts to declare.

## Supplementary Material

SC-013-D1SC06514A-s001

SC-013-D1SC06514A-s002
