# Supplementary material for: Kinetic trapping of a cobalt(ii) metallocage using a carbazole-containing expanded carbaporphyrinoid ligand
Source: Chem Sci. 2021 Dec 20;13(3):692–7. doi: 10.1039/d1sc06514a (PMC8768885; doi:10.1039/d1sc06514a)
Supplement: SC-013-D1SC06514A-s001 [file SC-013-D1SC06514A-s001.pdf]

Electronic Supplementary Material (ESI) for Chemical Science.
This journal is © The Royal Society of Chemistry 2021

Kinetic Trapping of a Cobalt(II) Metallocage using an Carbazole-Containing Expanded Carbaporphyrinoid Ligand

Weinan Zhou, Tridib Sarma, Yonghuan Su, Chuanhu Lei, and Jonathan L. Sessler

Table of Content

1. General Information S3

2. Synthetic Procedures and Compound Data S4

3. NMR and MS spectra S7

4. Crystal Data S15

5. Metalloring and Metallocage Crystallization Experimental S20

6. Optical and Electrochemical Properties S22

7. Magnetic Susceptibility Measurements S26

8. DFT Calculation S27

9. Supporting References S35

1. General Information

Reagents and instrumentations

All reagents and solvents were of commercial reagent grade and were used without further purification except where noted. Alumina column chromatography was performed on Merck deactivated Brockmann III neutral alumina oxide. Thin-layer chromatography (TLC) was carried out on pre-coated, glass-backed silica gel plates. All NMR spectra were acquired on either a Bruker 600 MHz instrument or a JEOL ECZ 400 MHz NMR spectrometer. Chemical shifts are reported in ppm using residual solvent signals as the internal reference standards. Complex ^{1}H NMR signals were assigned from ^{1}H-^{1}H COSY and 2D NOESY experiments. Spectroscopic solvents were purchased from Cambridge Isotope Laboratories. Mass spectra (MS) were recorded on a Bruker Daltonics Autoflex MALDI-TOF MS or HR-ESI-FT-ICR MS spectrometers. Cyclic voltammetric (CV) and differential pulse voltammetric (DPV) studies were carried out on a CH Instruments CHI660E potentiostat with an electrochemical system involving a three-electrode configuration consisting of a glassy carbon (working electrode), platinum wire (counter electrode) and a Ag/Ag^{+} (reference electrode) in dry dichloromethane with *n*-tetrabutylammonium hexafluorophosphate ($TBAPF_6$) as a supporting electrolyte. All solutions were degassed by means of sonication prior to their use in the electrochemical studies. Optical absorption spectra were recorded on a Varian Cary 5000 spectrophotometer. Magnetic susceptibility measurements were performed using a Quantum Design MPMS3 SQUID magnetometer. The χ value of **3·2Co** (5 mg) in a capsule was obtained from studies conducted over a temperature range from 300 to 2 K under a 1 T magnetic field. Each raw data file for the measured magnetic moment was corrected for the diamagnetic contribution of the sample holder and the Teflon bucket. Likewise, the molar susceptibility data was corrected for the diamagnetic contribution from the sample holder and the core diamagnetism (estimated using Pascal's constants[1]). The surface morphology and structure of {**3·2Co**}$_3$ and **3·2Co** were observed using scanning electron microscopy (SEM) without any sputter coating. X-ray single crystal diffraction analyses were performed on a Bruker SMART APEX diffractometer equipped with a CCD area detector using a graphite monochromator with CuK_α (λ = 1.54187 Å) or MoK_α (λ = 0.71073 Å) radiation. The structures were solved by direct method, followed by full-matrix least-squares refinement against F^2 using the SHELXS and SHELXL programs in the Olex2 package.[2] The crystal data has been deposited in the Cambridge Crystallographic Data Centre with reference nos. CCDC 2105486 [{**3·2Co**}$_3$], CCDC 2105487 [**3·2Zn**], CCDC 2105488 [**3·2Co**], and CCDC 2105812 [**3·2Pd**]. Crystallographic data are also listed in Tables S1 and S2.

2. Synthetic Procedures and Compound Data

Ligand **3** was prepared according to a previous procedure.[3]

Synthetic pathway leading to the metalation of ligand 3

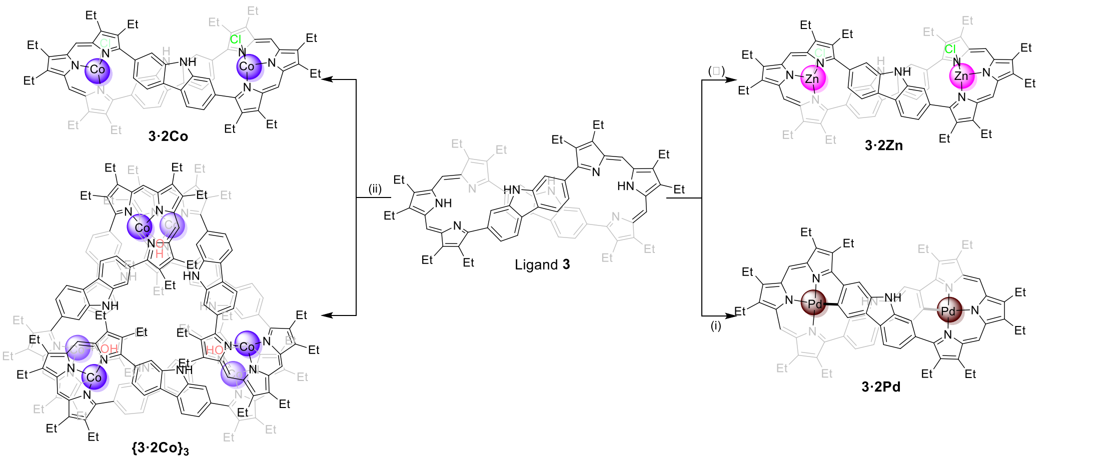

Scheme S1. Reagents and conditions: (i) $Pd(OAc)_2$, NaOAc, $CHCl_3$/MeOH, RT; (ii) $Co(OAc)_2 \cdot 4H_2O$, NaOAc, $CHCl_3$/MeOH, RT; (iii) $Zn(OAc)_2$, NaOAc, $CHCl_3$/MeOH, RT.

Synthesis of 3·2Pd

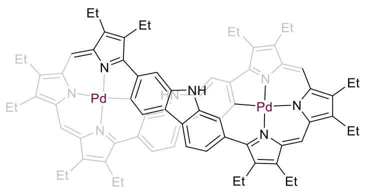

General method for the synthesis of 3·2Pd

A 100 mL two-necked round bottom flask equipped with a magnetic stirrer was charged with compound **3** (20 mg, 0.018 mmol), palladium acetate (40 mg, 0.18 mmol) and anhydrous sodium acetate (15 mg, 0.18 mmol). A mixture of 16 mL degassed $CHCl_3$ and 4 mL degassed MeOH was added *via* syringe under an inert atmosphere. The reaction mixture was then stirred for 8 hours at room temperature. After removal of the volatiles using a rotary evaporator, the residual was purified through a neutral alumina column using a mixture of CH_2Cl_2 and *n*-hexane (1:1) as the eluent. The brown-coloured fraction was collected and evaporated to dryness to give the desired product **3·2Pd**. Recrystallization from CH_2Cl_2/MeOH afforded a brown solid in 68% yield.

Analytical data for 3·2Pd: ^{1}H NMR (600 MHz, CD_2Cl_2, δ in ppm) δ 7.95 (d, J = 8.2, 2H), 7.78 (d, J = 8.2 Hz, 2H), 7.50 (s, 2H), 7.26 (s, 2H), 7.24 (s, 2H), 7.13 (s, 2H), 6.80 (s, 2H), 5.97 (s, 2H), 2.97 – 2.82 (m, 12H), 2.78 – 2.66 (m, 12H), 1.40 – 1.36 (m, 12H), 1.32 – 1.29 (m, 12H), 1.27 – 1.22 (m, 12H). ^{13}C NMR (150 MHz, CD_2Cl_2, δ in ppm) δ 173.89, 171.57, 157.78, 150.54, 148.11, 143.84, 142.61, 142.45, 141.98, 140.45, 140.30, 139.26, 138.72, 138.49, 134.26, 133.71, 131.16, 127.45, 124.99, 120.86, 120.59, 120.15, 119.94, 118.52, 117.53, 106.95, 19.78, 19.00, 18.96, 18.17, 18.10, 17.98, 17.65, 17.50, 17.38, 17.20, 15.68, 15.12.

MS (MALDI-TOF) *m/z*: Calcd for $C_{76}H_{76}N_8Pd_2$ = 1314.4267 $[M]^+$; found = 1314.3255.

UV-Vis (CH_2Cl_2): λ_{max} [nm] (ε $[M^{-1}\ cm^{-1}]$ x10^5) = 481 (0.64), 818 (0.10).

Synthesis of 3·2Co

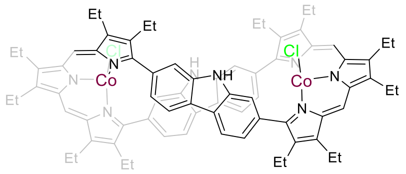

General method for the synthesis of 3·2Co

A 100 mL two-necked round bottom flask equipped with magnetic stirrer was charged with ligand **3** (20 mg, 0.018 mmol), cobalt acetate tetrahydrate (45 mg, 0.18 mmol) and sodium acetate anhydrous (15 mg, 0.18 mmol). A solvent mixture consisting of 16 mL degassed $CHCl_3$ and 4 mL degassed MeOH was added via syringe under an inert atmosphere and the reaction was stirred for 8 hours. After removal of the volatiles using a rotary evaporator, the residual was purified through a neutral alumina column using a mixture of MeOH/CH_2Cl_2 (3%) as the eluent. The olive-coloured fraction was collected and evaporated to dryness to give the desired metallic product. Recrystallization from supersaturated CH_2Cl_2/*n*-hexane afforded a metallic cyan solid in the form of needles in 85% yield. Note: Recrystallize slowly from unsaturated $CHCl_3$/*n*-hexane afforded a block dark cyan solid consisting of **{3·2Co}**$_3$.

Analytical data for 3·2Co: ^{1}H NMR (600 MHz, $CDCl_3$, δ in ppm) δ 49.88, 47.74, 47.69, 46.07, 44.75, 21.56, 19.06, 17.51, 16.45, 13.57, 12.10, 9.49, 7.93, 7.43, 4.25, -4.37, -5.93, -6.40, -8.96, -14.32, -24.05.

MS (MALDI-TOF) *m/z*: Calcd for $C_{76}H_{78}N_8ClCo_2$ = 1255.4697 $[M-Cl]^+$; found = 1255.3670.

UV-Vis (CH_2Cl_2): λ_{max} [nm] (ε $[M^{-1}\ cm^{-1}]$ x10^5) = 463 (0.79), 725 (0.33).

Synthesis of 3·2Zn

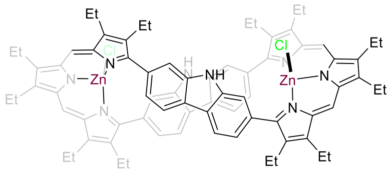

General method for the synthesis of 3·2Zn

A 100 mL two-necked round bottom flask equipped with magnetic stirrer was charged with compound **3** (20 mg, 0.018 mmol), anhydrous zinc acetate (33 mg, 0.18 mmol) and anhydrous sodium acetate (15 mg, 0.18 mmol). A solvent mixture consisting of 16 mL degassed $CHCl_3$ and 4 mL degassed MeOH was added via syringe under an inert atmosphere. The reaction mixture was then stirred for 12 hours at room temperature. After removal of the volatiles using a rotary evaporator, the residual was purified through a neutral alumina column using a mixture MeOH/CH_2Cl_2 (2%) as the eluent. The olive-coloured fraction was collected and evaporated to dryness to give the desired product **3·2Zn**. Recrystallization from CH_2Cl_2/MeOH afforded a metallic dark purple solid in the form of needle-like crystals in 86% yield.

Analytical data for 3·2Zn: ^{1}H NMR (400 MHz, CD_2Cl_2, -60 °C, δ in ppm) δ 8.50 (s, 2H), 8.22 (d, J = 8.2 Hz, 2H), 8.10 (s, 2H), 7.90 (d, J = 8.1 Hz, 2H), 7.64 (s, 2H), 7.35 (d, J = 8.7 Hz, 4H), 6.85 (d, J = 8.1 Hz, 2H), 6.15 – 6.05 (m, 2H), 2.95 – 2.59 (m, 24H), 1.36 – 1.20 (m, 36H).

MS (MALDI-TOF) *m/z*: Calcd for $C_{76}H_{79}N_8Zn_2$ = 1233.4975 [M -2Cl + H]$^+$; found = 1233.3783.

UV-Vis (CH_2Cl_2): λ_{max} [nm] (ε [M^{-1} cm^{-1}] x10^5) = 461 (0.93), 702 (0.35).

3. NMR and MS spectra

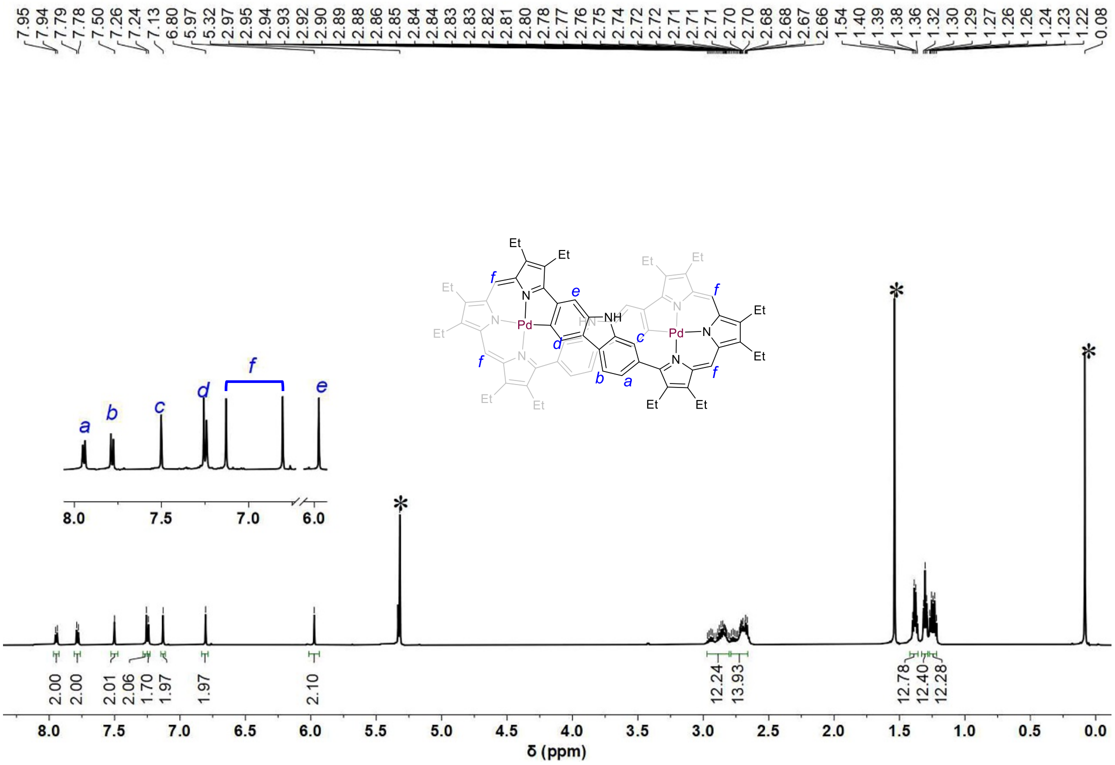

Figure S1. ^{1}H NMR spectrum of **3·2Pd** recorded in CD_2Cl_2 at 25 °C. *Asterisk indicates residual solvent impurities.

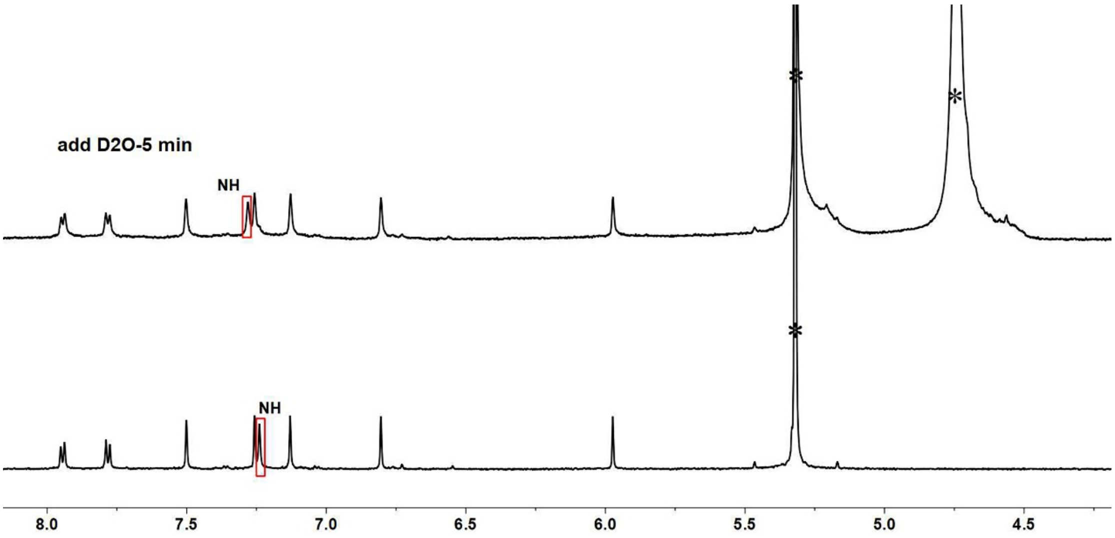

Figure S2. Comparative ^{1}H NMR spectra of **3·2Pd** before (bottom) and after (top) the addition of D_2O recorded in CD_2Cl_2 at 25 °C. *Asterisk indicates residual solvent impurities.

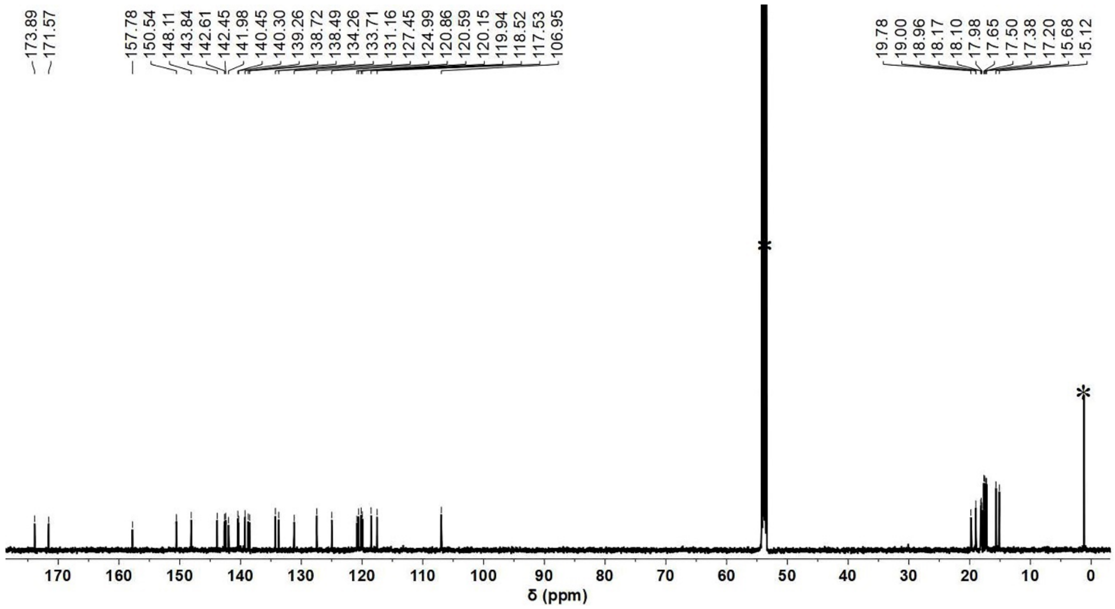

Figure S3. ^{13}C NMR spectrum of **3·2Pd** recorded in CD_2Cl_2 at 25 °C. *Asterisk indicates residual solvent impurities.

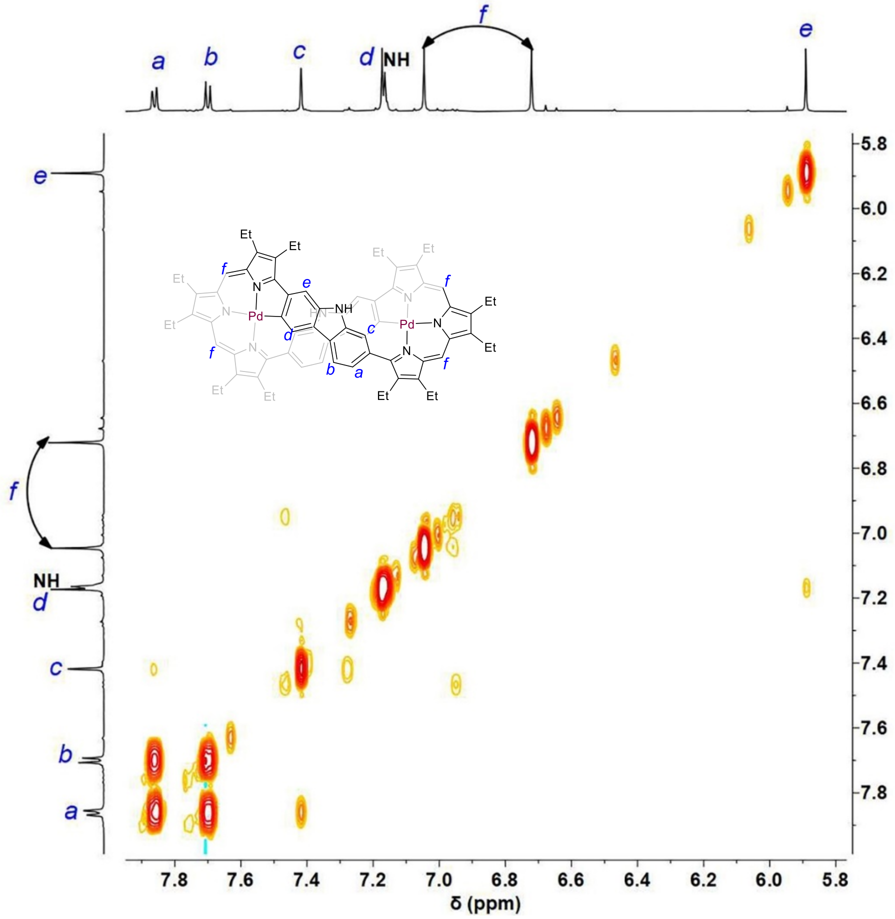

Figure S4. ^{1}H-^{1}H COSY spectrum of **3·2Pd** (aromatic region) recorded in CD_2Cl_2 at 25 °C.

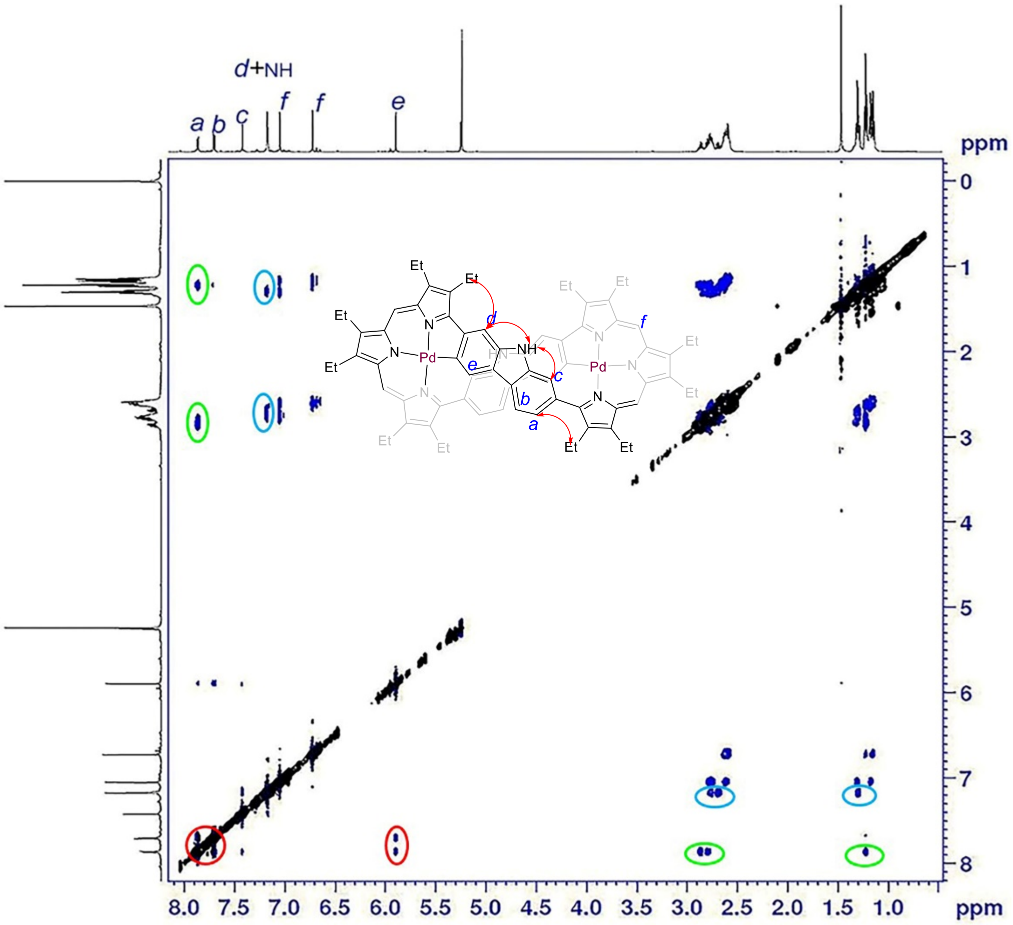

Figure S5. NOESY spectrum of **3·2Pd** recorded in CD_2Cl_2 at 25 °C. Strong NOE effects are highlighted using double arrows or circles.

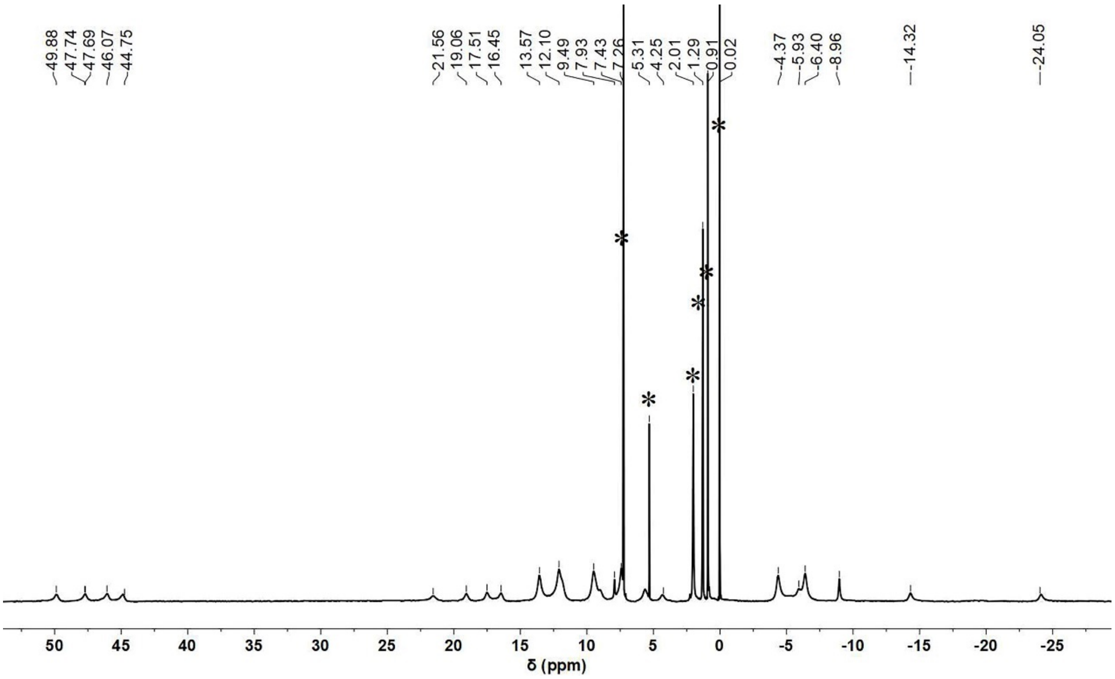

Figure S6. ^{1}H NMR spectrum of **3·2Co** recorded in $CDCl_3$ at 25 °C. *Asterisk indicates residual solvent impurities.

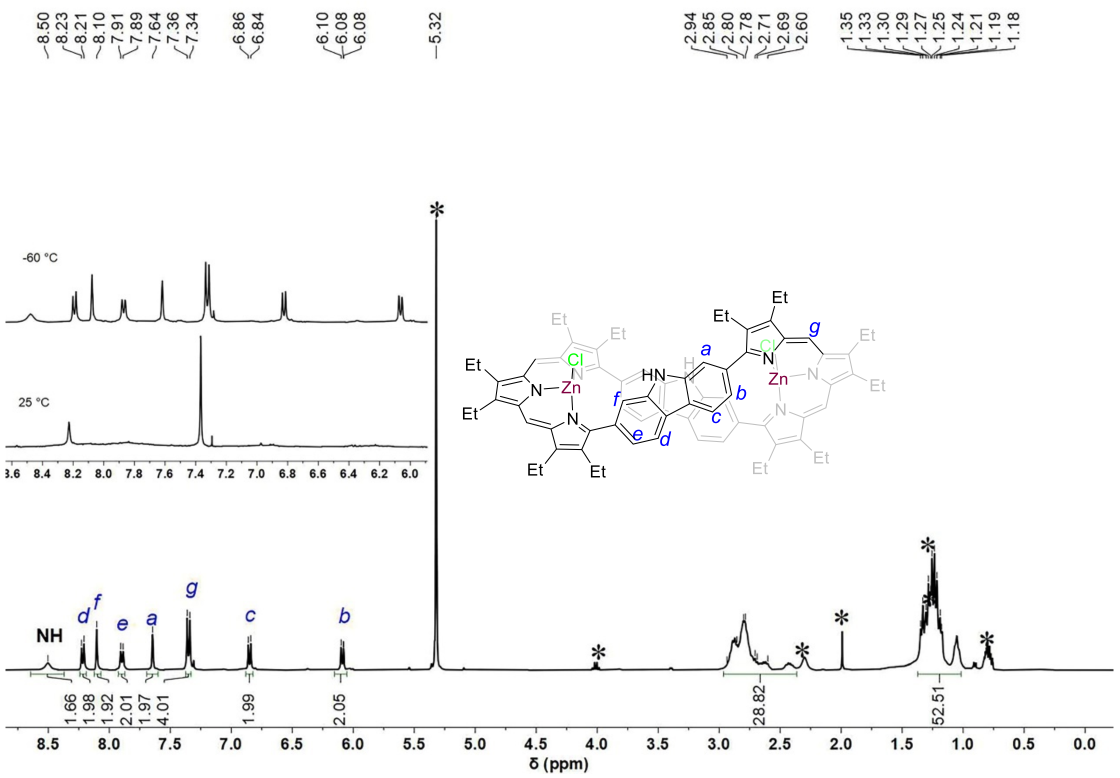

Figure S7. ^{1}H NMR spectrum of **3·2Zn** recorded in CD_2Cl_2 at -60 °C. Insert: comparative spectra of **3·2Zn** at 25 °C and -60 °C. *Asterisk indicates residual solvent impurities.

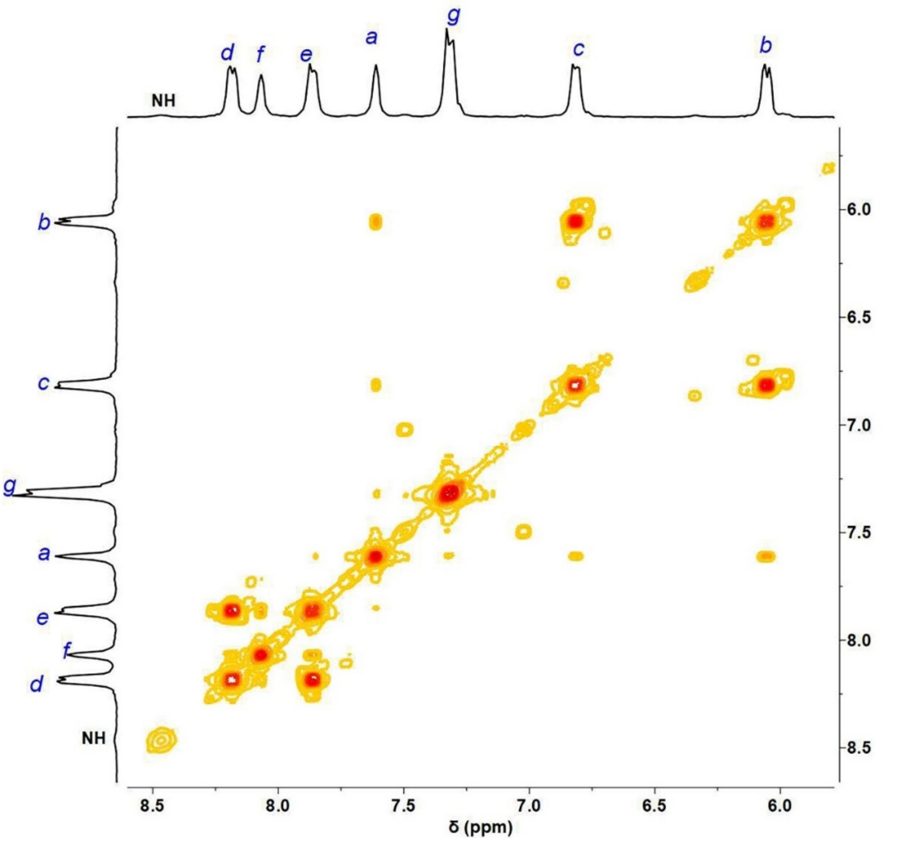

Figure S8. ^{1}H-^{1}H COSY spectrum of **3·2Zn** (aromatic region) recorded in CD_2Cl_2 at -60 °C.

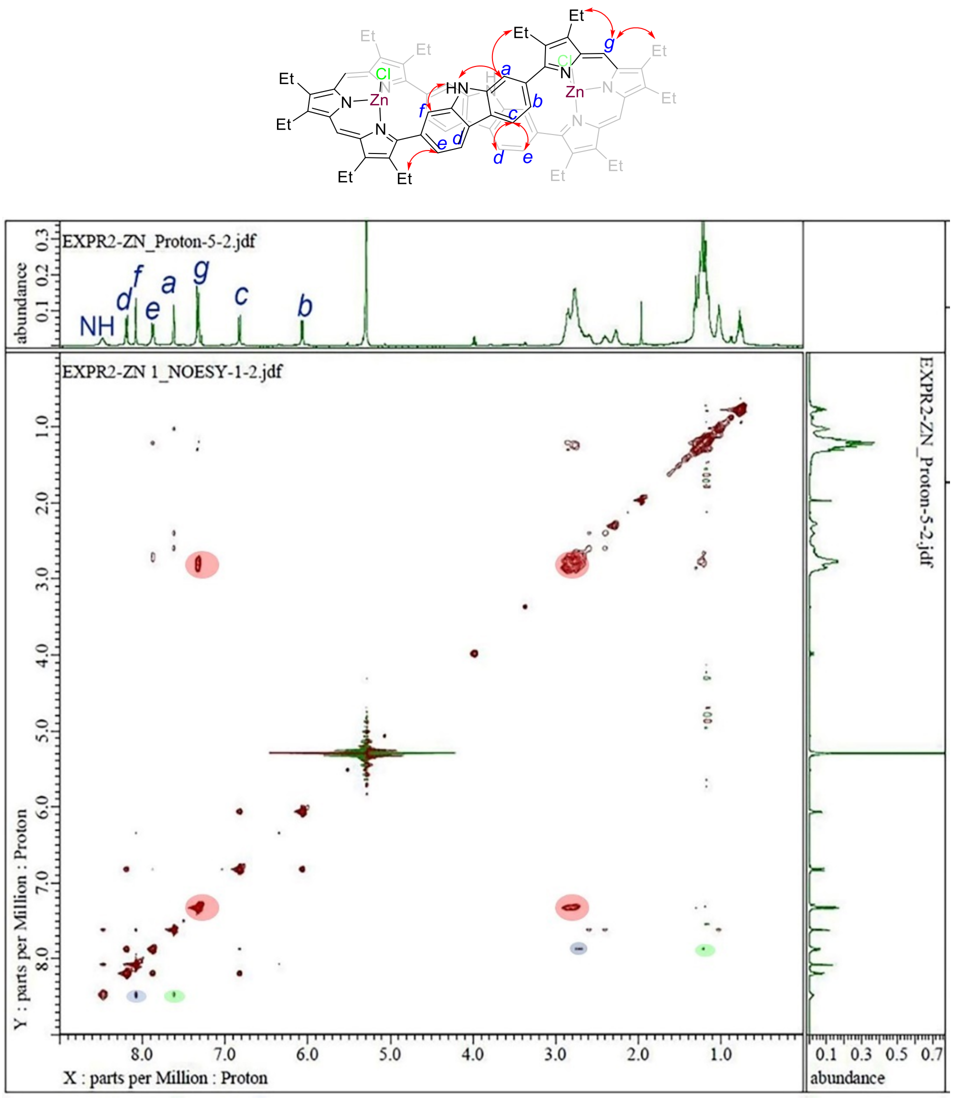

Figure S9. NOESY spectrum of **3·2Zn** recorded in CD_2Cl_2 at -60 °C. NOE effects are highlighted in red or with double arrows.

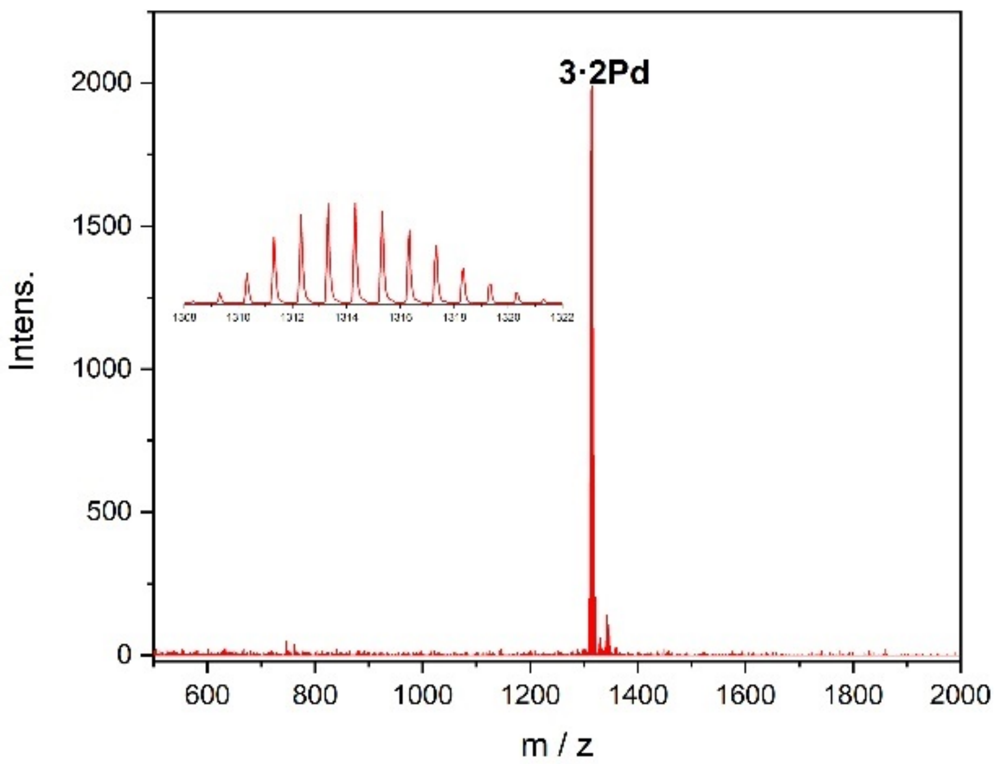

Figure S10. Observed MALDI-TOF MS spectrum of **3·2Pd** in THF.

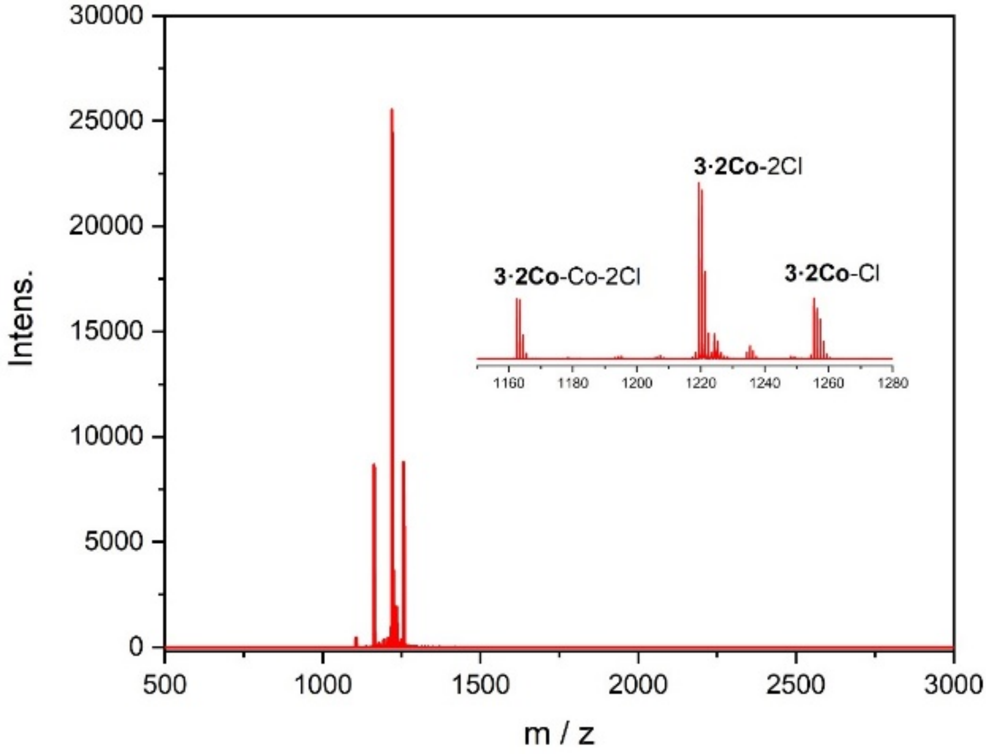

Figure S11. Observed MALDI-TOF MS spectrum of **3·2Co** in THF.

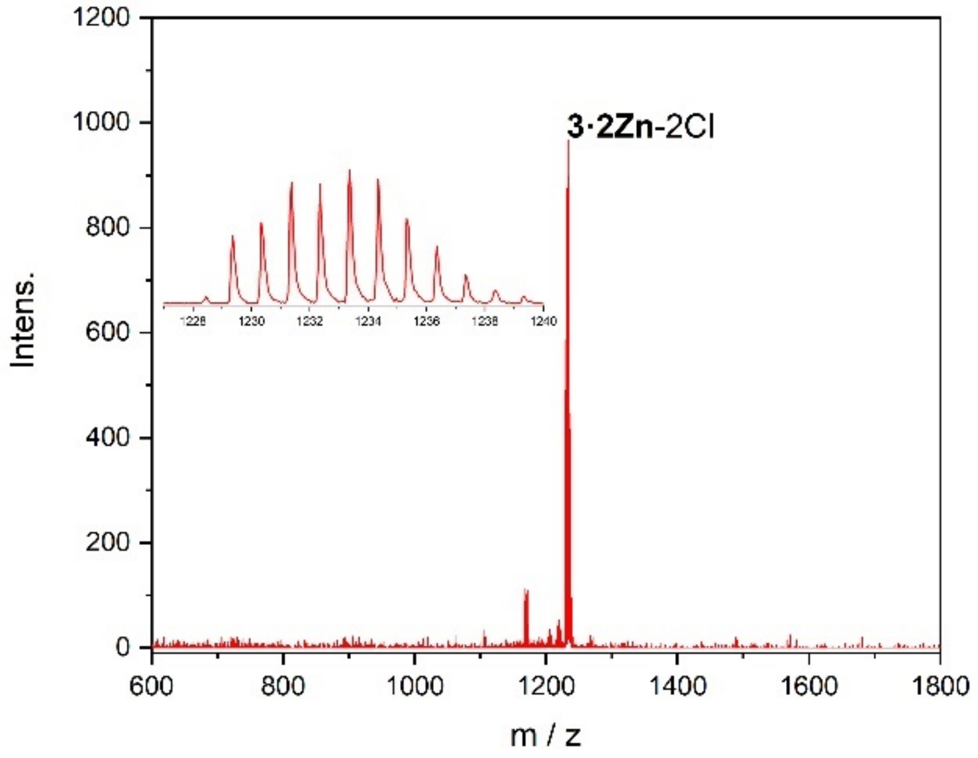

Figure S12. Observed MALDI-TOF MS spectrum of **3·2Zn** in THF.

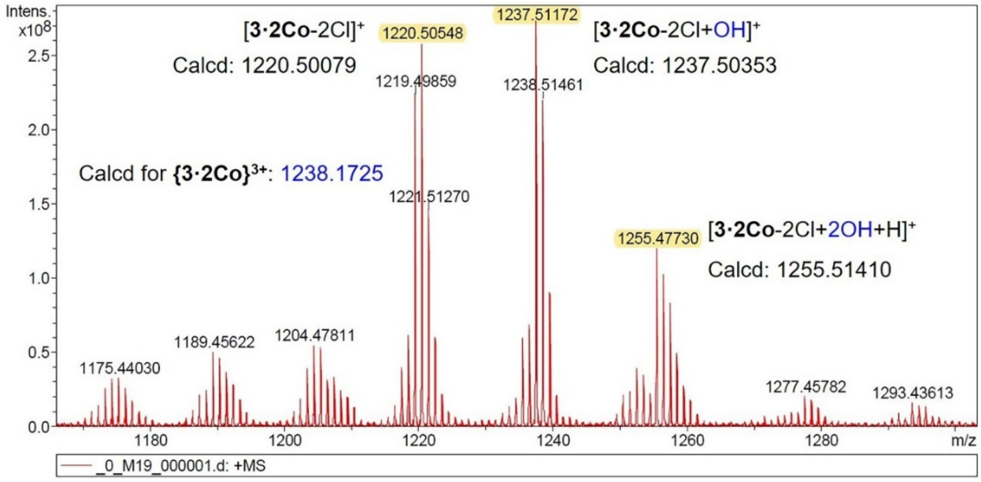

Figure S13. Observed and calculated HR-FT-ICR MS spectrum of {**3·2Co**}$_3$ in acetonitrile.

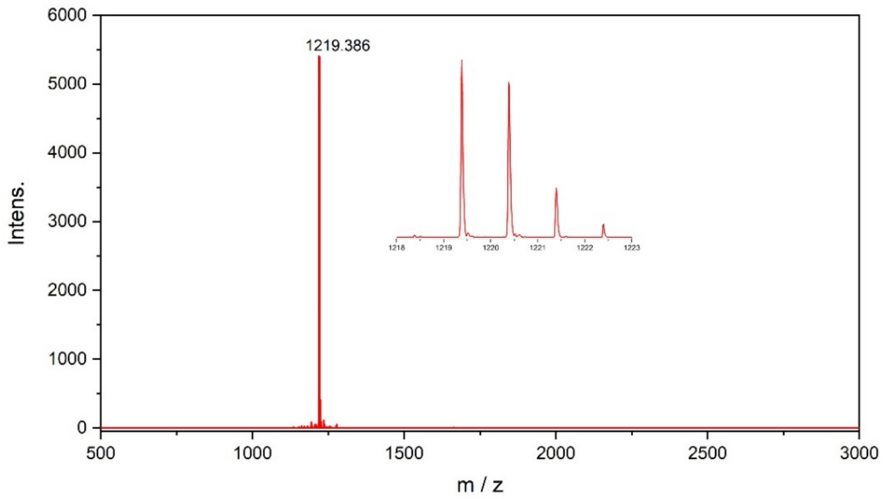

Figure S14. Observed MALDI-TOF MS spectrum of {**3·2Co**}$_3$ after heating at reflux for 12 h in THF.

4. Crystal Data

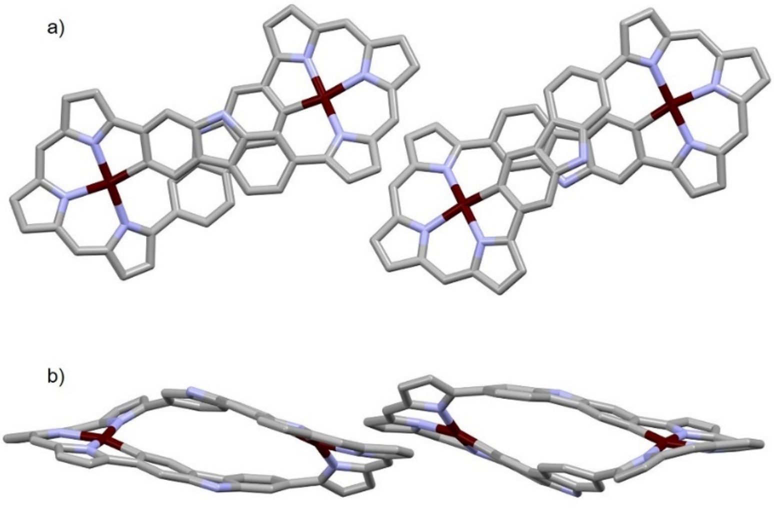

Figure S15. X-Ray crystal structure of **3·2Pd**; a) top view, b) side view. Peripheral alkyl groups and hydrogen atoms have been omitted for clarity.

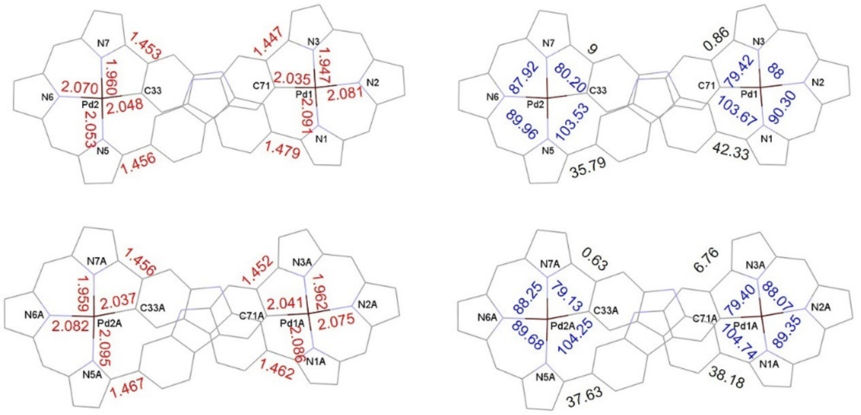

Figure S16. Selected bond lengths in Å (numbers in red) and bond angles in degrees (numbers in blue) and torsion angles (numbers in black), again in degrees, defined by the tripyrrin segments and carbazole units of the two different structures of **3·2Pd** seen in the single crystal subject to X-ray diffraction analysis. Peripheral alkyl groups and hydrogen atoms have been omitted for clarity.

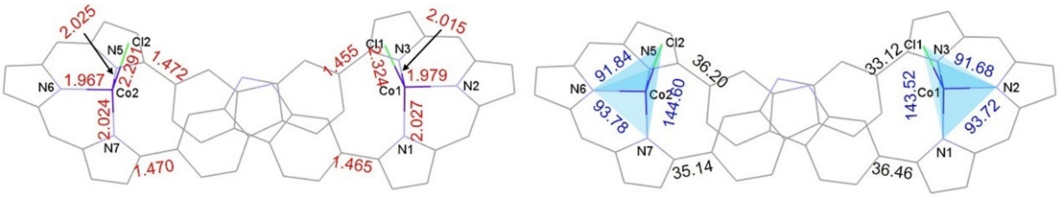

Figure S17. Selected bond lengths in Å (numbers in red) and bond angles (numbers in blue) in degrees and torsion angles, also in degrees, (numbers in black) defined by tripyrrin segments and the carbazole units as obtained from the single crystal X-ray structure of **3·2Co**. Peripheral alkyl groups and hydrogen atoms have been omitted for clarity.

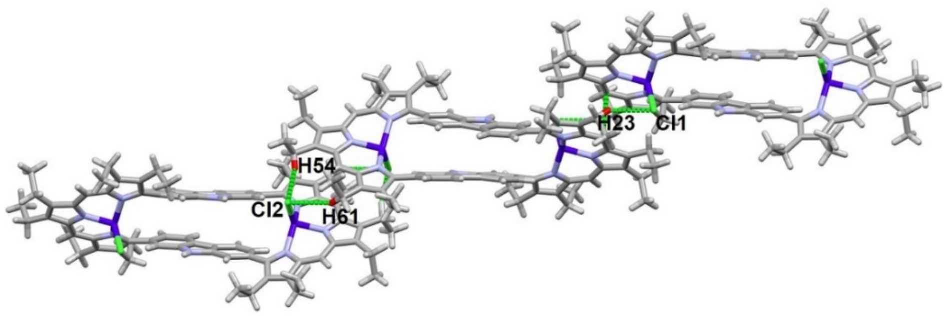

Figure S18. Single crystal X-ray structure of **3·2Co** shown as a 1-D array. The bond distances of Cl1…H23: 2.907 Å, Cl2…H54: 2.843 Å, Cl2…H61: 2.877 Å.

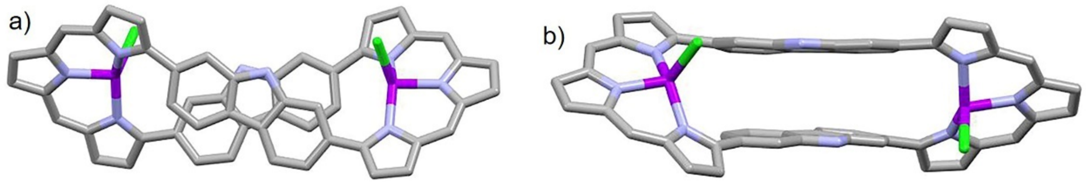

Figure S19. X-Ray crystal structure of **3·2Zn**; a) top view, b) side view. Peripheral alkyl groups and hydrogen atoms have been omitted for clarity.

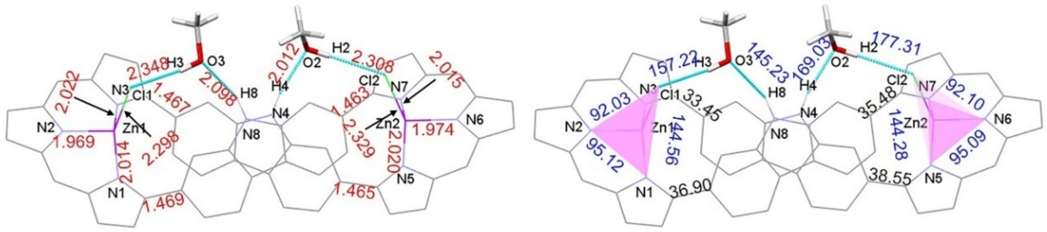

Figure S20. Selected bond lengths in Å (numbers in red) and bond angles in degrees (numbers in blue) and torsion angles (numbers in black), also in degrees, defined by tripyrrin segments and carbazole units as obtained from the single crystal X-ray structure of **3·2Zn**. Peripheral alkyl groups and hydrogen atoms were omitted for clarity.

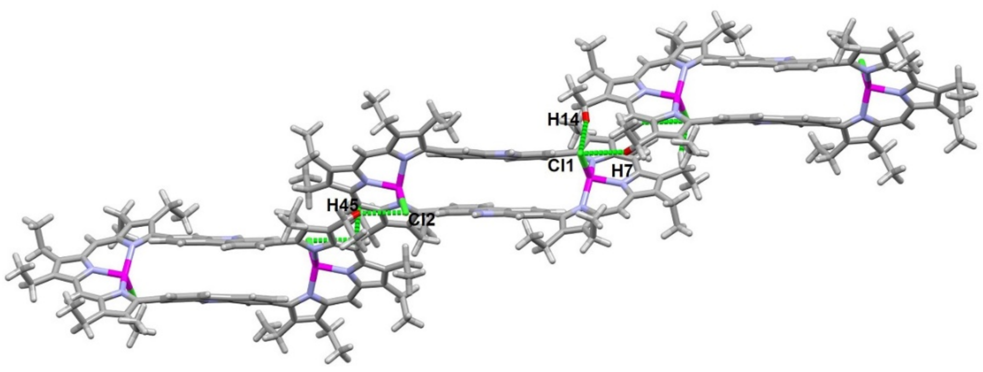

Figure S21. Single crystal X-ray structure of **3·2Zn** shown as a 1-D array. Selected bond distances: Cl1…H7: 2.889 Å, Cl1…H14: 2.739 Å, Cl2…H45: 2.856 Å.

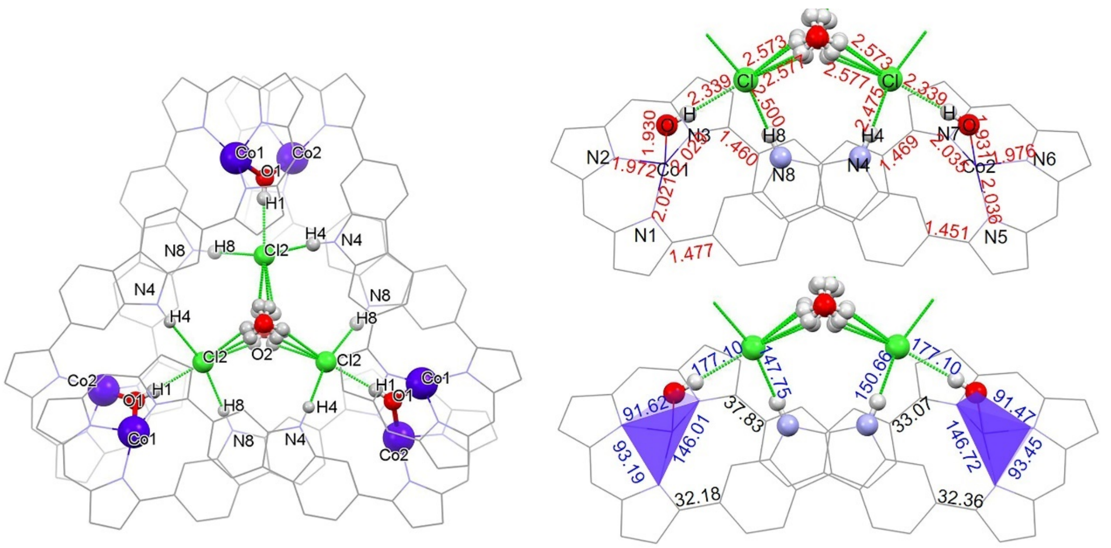

Figure S22. Selected bond lengths (numbers in red) in Å and bond angles (numbers in blue) in degrees and torsion angles (numbers in black), also in degrees, defined by the tripyrrin segments and the carbazole units as obtained from the single crystal X-ray structure of **{3·2Co}$_3$**. Peripheral alkyl groups and hydrogen atoms that are not involved in intermolecular interactions have been omitted for clarity.

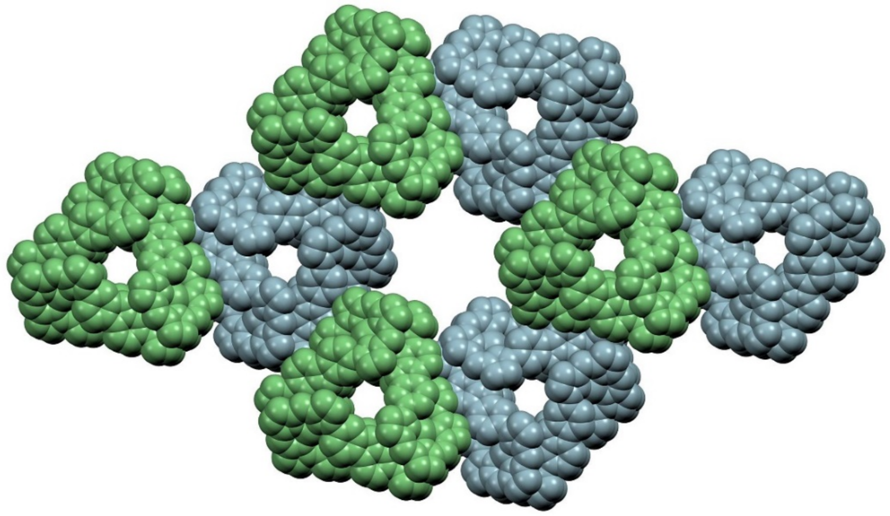

Figure S23. Single crystal X-ray packing structure of **{3·2Co}$_3$**. Peripheral alkyl groups and hydrogen atoms have been omitted for clarity.

Table S1. Crystal data and structure refinements for **3·2Pd** and **3·2Zn**.

**Compound**	**3·2Pd**	**3·2Zn**
Formula	$2(C_{76}H_{76}N_8Pd_2)$	$C_{76}H_{78}Cl_2N_8Zn_2$, $2(CHCl_3)$, $3(CH_4O)$
M_r	2628.49	1639.96
Temperature/K	150.0	173.0
Crystal system	Triclinic	Monoclinic
Space group	P-1<br>2	$P2_1/c$<br>14
a/Å	11.6940(4)	28.535(6)
b/Å	20.7153(6)	13.861(3)
c/Å	27.7180(9)	20.682(4)
α/°[deg]	68.590(1)	90
β/°[deg]	89.019(2)	101.36(3)
γ/°[deg]	82.809(2)	90
Volume[Å3]	6199.0(3)	8020(3)
Z	2	4
ρcalc[g/cm^3]	1.408	1.358
Radiation	CuKα (λ = 1.54178)	MoKα (λ = 0.71073)
Reflections collected	132146	28063
Goodness-of-fit on F^2	1.044	1.081
Final R indices [I>=2σ (I)]	R1 = 0.0522, wR2 = 0.1422	R1 = 0.0931, wR2 = 0.2829
R indices [all data]	R1 = 0.0579, wR2 = 0.1480	R1 = 0.1050, wR_2 = 0.3013
CCDC no.	2105812	2105487
Solvents	CH_2Cl_2/MeOH	$CHCl_3$/MeOH

Table S2. Crystal data and structure refinements for **3·2Co** and {**3·2Co**}$_3$.

**Compound**	**3·2Co**	**{3·2Co}$_3$**
Formula	$C_{76}H_{78}Cl_2Co_2N_8$, CH_2Cl_2	$C_{76}H_{79}Co_2N_8O$, 1.167(Cl), $CHCl_3$, 0.667(H_2O)
M_r	1339.41	1411.06
Temperature/K	150.0	173.0
Crystal system	monoclinic	trigonal
Space group	$P2_1/c$ 13	P-3 147
a/Å	28.3373(6)	31.1312(4)
b/Å	14.5574(3)	31.1312(4)
c/Å	20.3283(5)	15.5307(3)
α/°[deg]	90	90
β/°[deg]	100.895(1)	90
γ/°[deg]	90	120
Volume[Å^3]	8234.6(2)	13035.1(4)
Z	4	6
ρcalc[g/cm^3]	1.111	1.079
Radiation	CuKα (λ = 1.54178)	CuKα (λ = 1.54178)
Reflections collected	50287	165078
Goodness-of-fit on F^2	1.060	1.033
Final R indices [I>=2σ (I)]	R1 = 0.0583, wR2 = 0.1701	R1 = 0.0632, wR2 = 0.1780
R indices [all data]	R1 = 0.0717, wR2 = 0.1810	R1 = 0.0830, wR2 = 0.1939
CCDC no.	2105488	2105486
Solvents	CH_2Cl_2/*n*-hexane	$CHCl_3$/*n*-hexane

5. Metalloring and Metallocage Crystallization Experimental

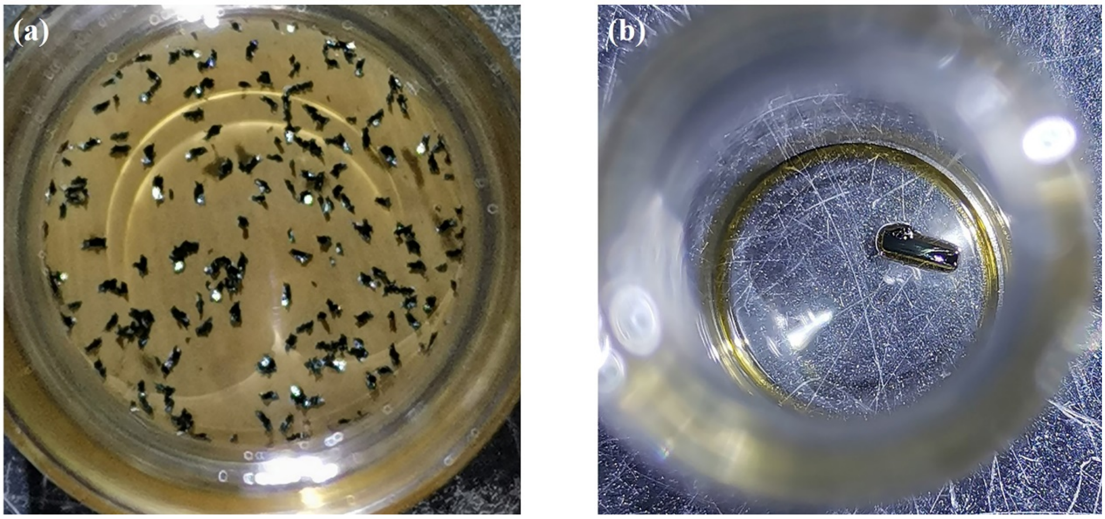

Figure S24. Photographs of the crystals of (a) **3·2Co** and (b) **{3·2Co}**$_3$. (Note: rhombic-like crystals of **3·2Co** were obtained by liquid diffusion of *n*-hexane into a supersaturated dichloromethane solution (CH_2Cl_2/*n*-hexane = 1:4, V/V) at room temperature in a sealed vial for 3-5 days; a brick-like crystal of **{3·2Co}**$_3$ was obtained by slow vapor diffusion of *n*-hexane into an unsaturated chloroform solution of the complex ($CHCl_3$/*n*-hexane = 1:2, V/V) at room temperature over the course of about three weeks.

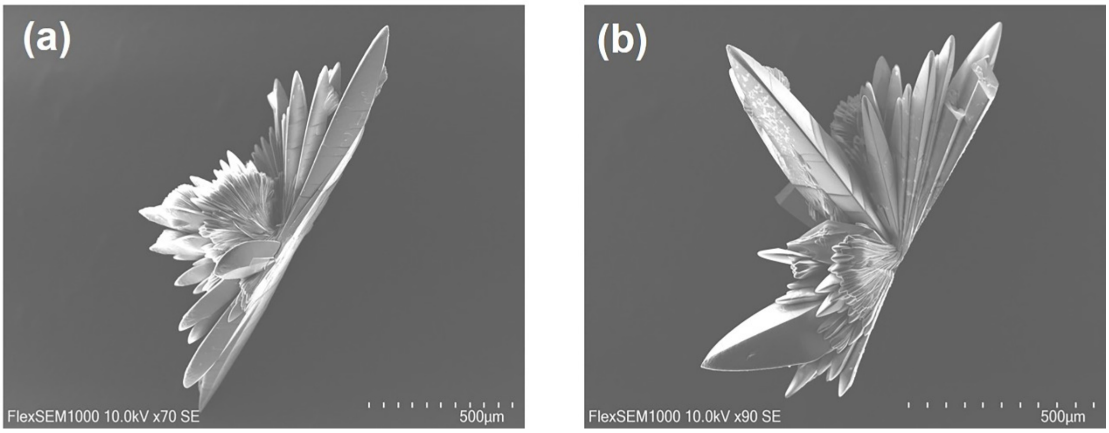

Figure S25. SEM images of **3·2Co**.

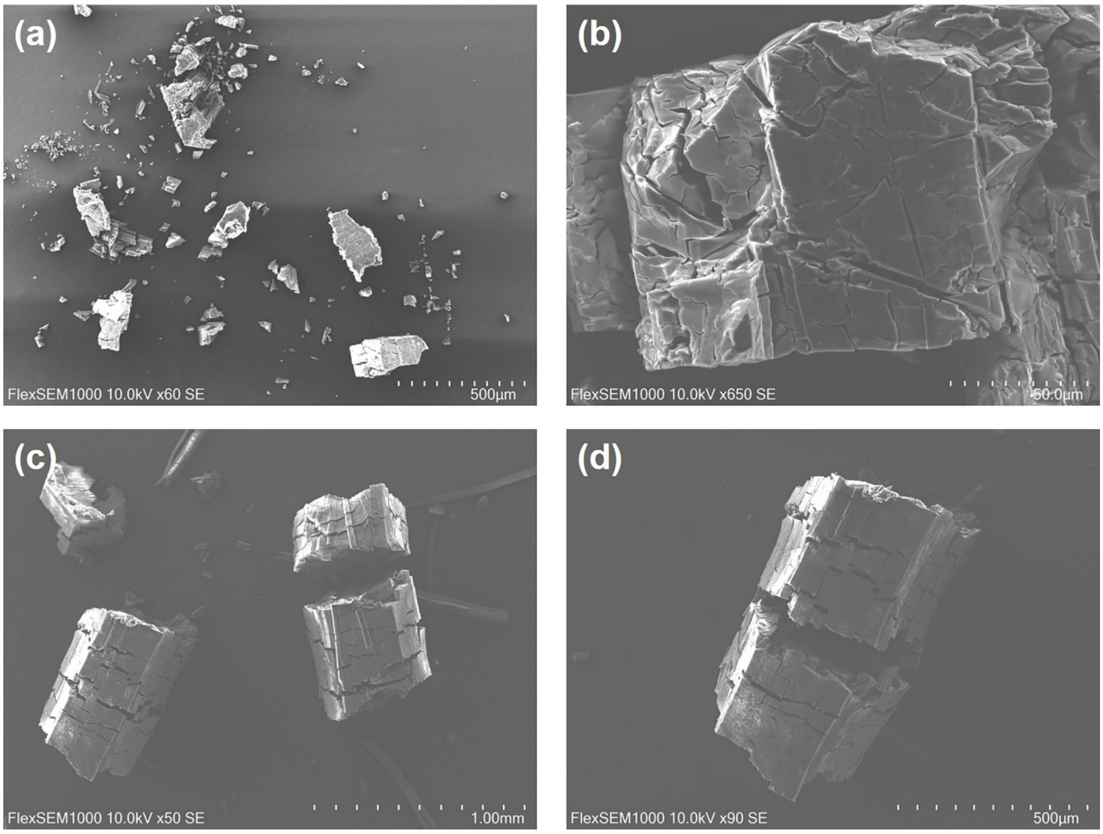

Figure S26. SEM images of **{3·2Co}**$_3$.

6. Optical and Electrochemical Properties

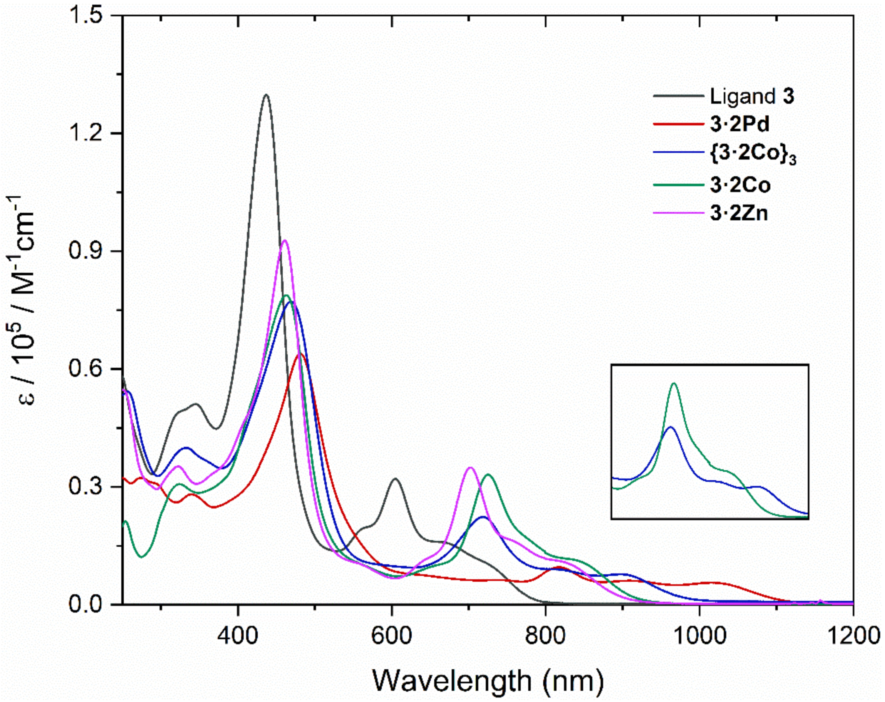

Figure S27. Absorption spectra of ligand **3**, **3·2Pd**, **{3·2Co}**$_3$, **3·2Co**, and **3·2Zn** recorded at room temperature in $CHCl_3$. Insert: Comparison of the spectra of **{3·2Co}**$_3$ and **3·2Co** within the expanded 600 – 1000 nm spectral region.

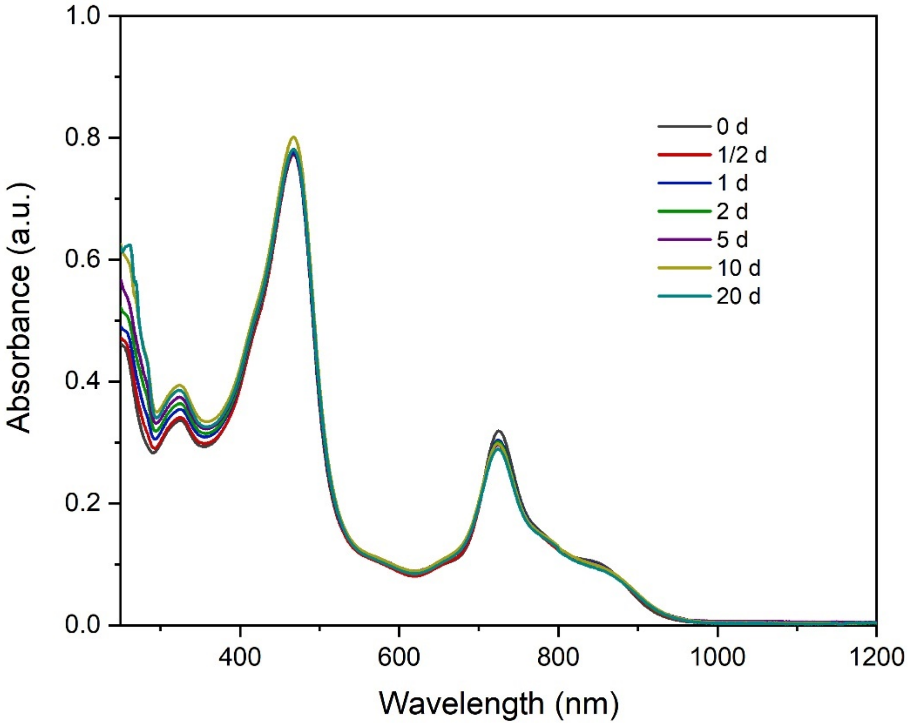

Figure S28. Absorption spectral changes seen for **3·2Co** at room temperature in $CHCl_3$ as a function of time. Here, d = days.

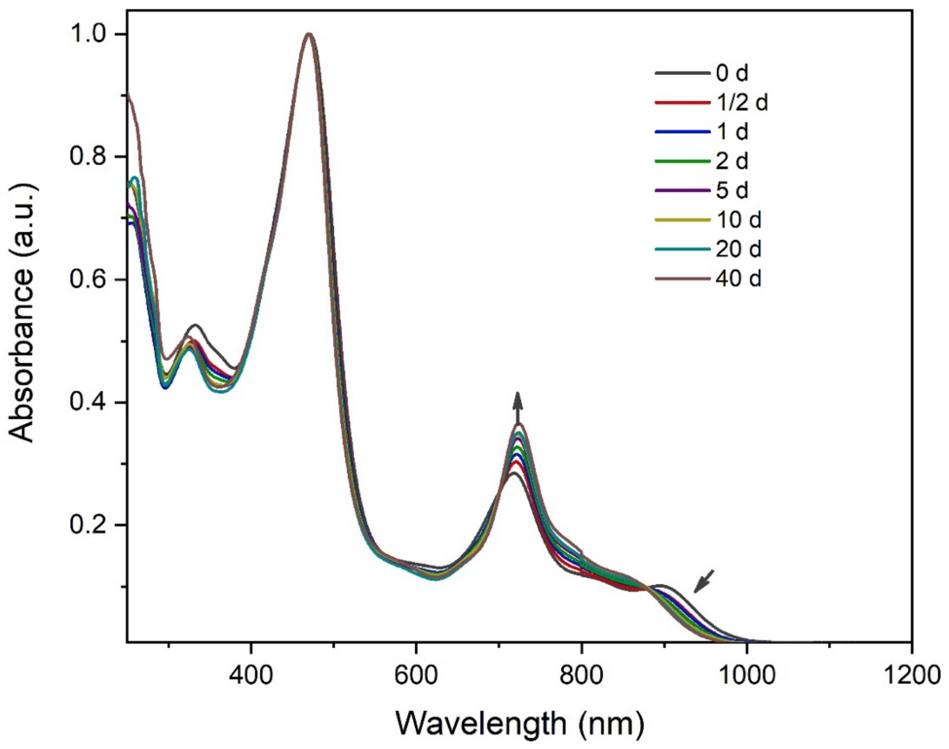

Figure S29. Normalized time-dependent change in the UV/vis-NIR absorption spectrum of **{3·2Co}**$_3$ recorded in $CHCl_3$ at room temperature. Here, d = days.

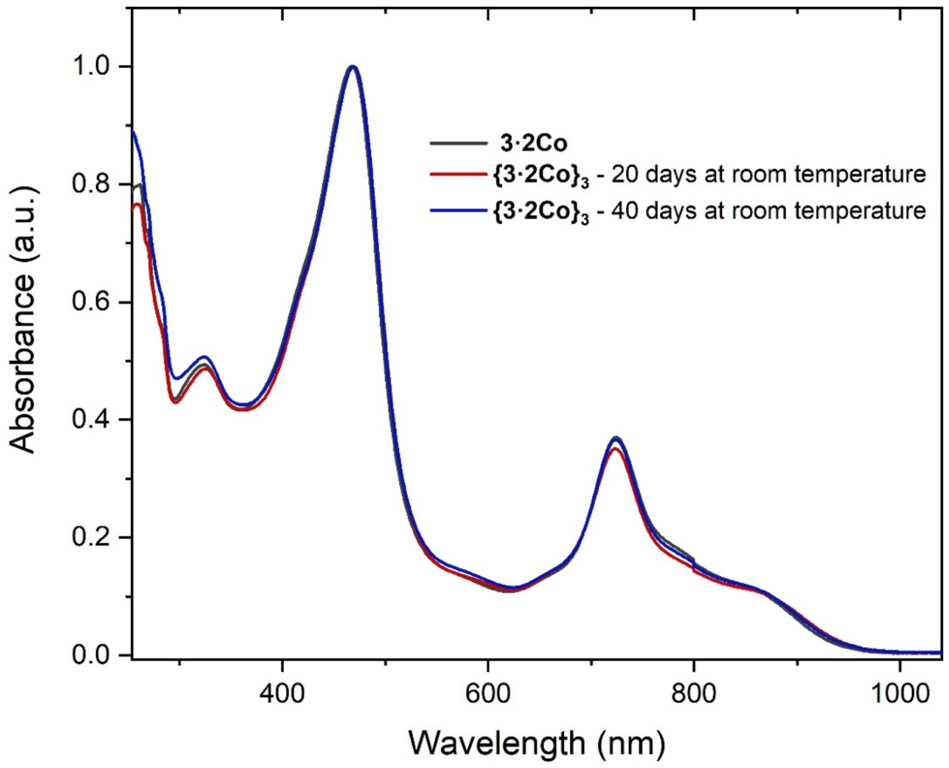

Figure S30. Comparative UV/vis-NIR absorption spectrum of **3·2Co** and **{3·2Co}**$_3$ recorded in $CHCl_3$ at room temperature.

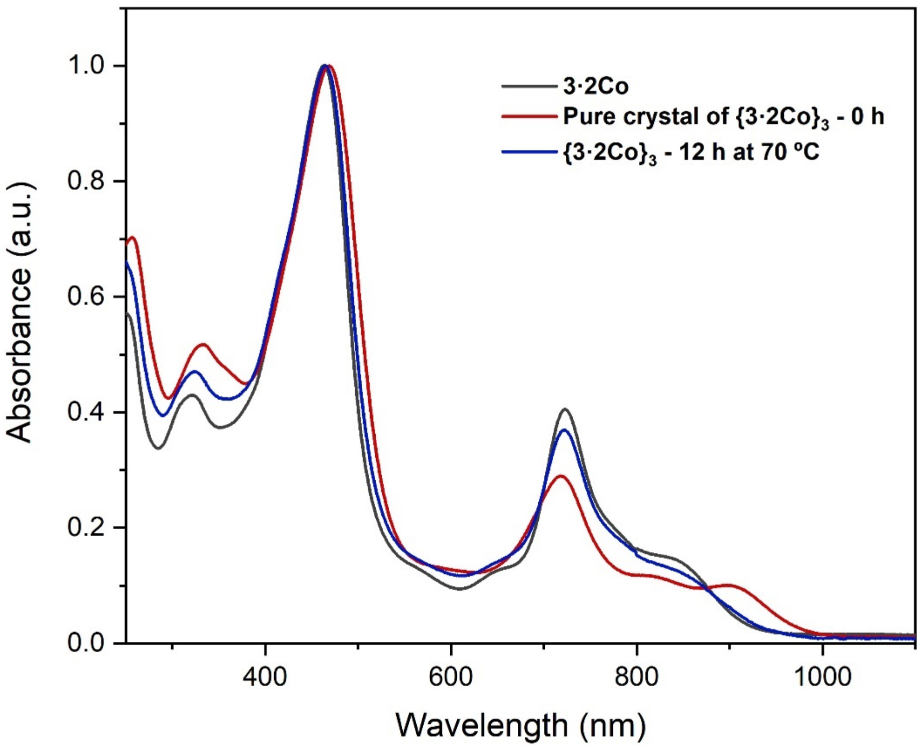

Figure S31. Normalized absorption spectra showing the changes in the optical features of **{3·2Co}**$_3$ recorded in $CHCl_3$ after subjecting to heating at reflux in $CHCl_3$ for 12 h.

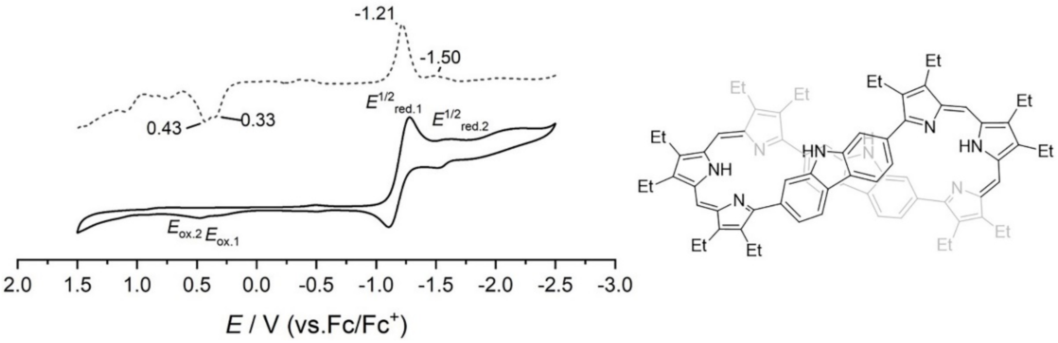

Figure S32. Cyclic voltammogram and differential pulse voltammogram of **3**.

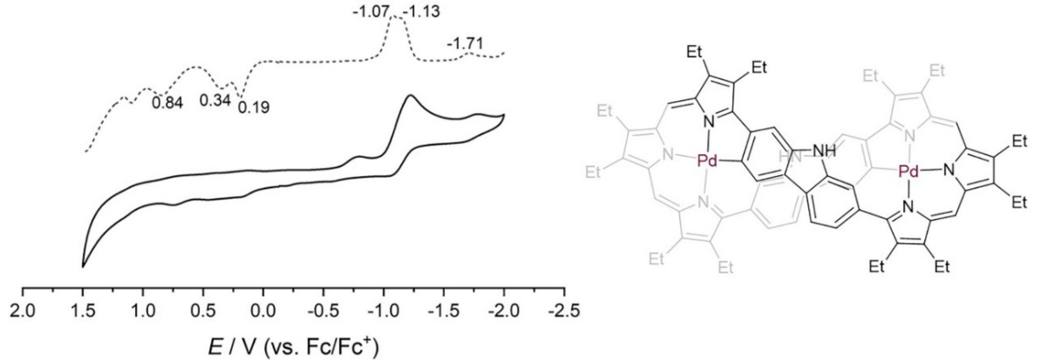

Figure S33. Cyclic voltammogram and differential pulse voltammogram of **3·2Pd**.

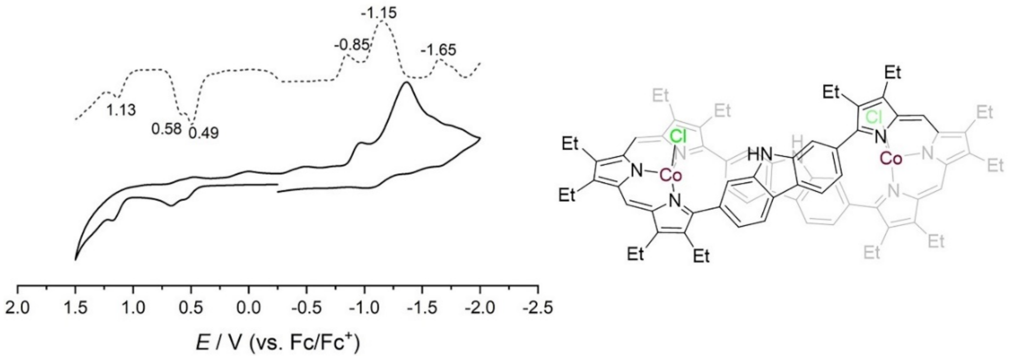

Figure S34. Cyclic voltammogram and differential pulse voltammogram of **3·2Co**.

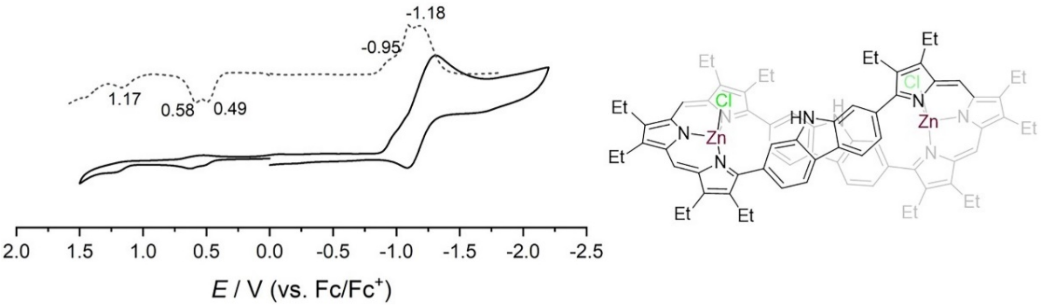

Figure S35. Cyclic voltammogram and differential pulse voltammogram of **3·2Zn**.

7. Magnetic Susceptibility Measurements

The variable-temperature magnetic susceptibility was measured over the temperature range of 2 to 300 K under an applied magnetic fields of 1 T by using a Quantum Design MPMS3 magnetometer. Figure S35 show a $\chi_M T$ v.s. T plot for the complex **3·2Co**. At 300 K, **3·2Co** exhibits a value of $\chi_M T$ = 5.21 cm^3 K mol^{-1} corresponding to two magnetically noninteracting S = 3/2 Co(II) centres. As the temperature is decreased, the value of $\chi_M T$ undergo a gradual then rapid decline, reaching minimum value of 2.48 cm^3 K mol^{-1} at 2 K, indicative of a weak intermolecular antiferromagnetic interactions. Considering the long intramolecular separation between the two Co(II) ions, only an intermolecular magnetic exchange interaction was considered. Therefore, the $\chi_M T$ v.s. T curve of complex **3·2Co** is fitted using a single-core model. The spin Hamiltonian is given in eq. (1). Red circles are the observed values and the solid line was fitted using the PHI[4] program. The fit gives the parameters, g = 2.57 and ZJ = -0.033 cm^{-1}.

$$\hat{H} = D\left[\hat{S}_z^2 - \frac{S(S+1)}{3}\right] + E\left(\hat{S}_x^2 - \hat{S}_y^2\right) + g\mu_B SH \quad (1)$$

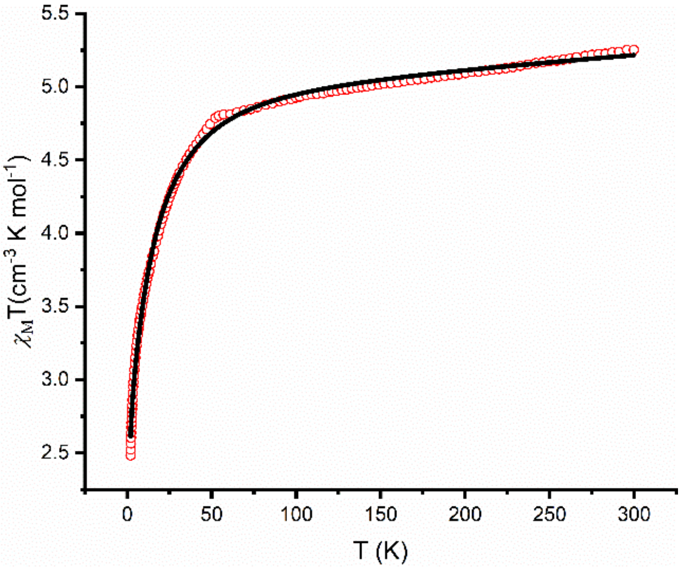

Figure S36. Variable-temperature χ_MT *vs.* T plots for **3·2Co** collected under an applied field of 1 T. The black line is the fit to the data, whereas the red circles are observed values.

8. DFT Calculation

All calculations were carried out using the Gaussian 16 program.[5] Initial geometries for **3·2Zn**, **3·2Pd**, **3·2Co** were obtained from the X-ray structures. The structures were fully optimized without any symmetry restrictions. Geometry optimizations in the ground state (S0) were performed using density functional theory (DFT) with Becke's three-parameter hybrid exchange functionals and the Lee-Yang-Parr correlation functional (B3LYP) employing the 6-311G(d, p) basis set for main group atoms and LANL2DZ for metal atoms. The spin singlet states of metal complexes **3·2Pd**, **3·2Co**, and **3·2Zn** were optimized in its ground (S0) state using the B3LYP/6-311G(d,p) and LANL2DZ levels.[6] At same time, the magnetic state (triplet state) of **3·2Co** was optimized at the UB3LYP level using the same basis sets. FMOs were obtained using Multiwfn 3.8 software.[7] Using the ORCA5.0 software[8], the time-dependent DFT (TD-DFT) method at the PBE0/Def2SVP[9] level was employed to simulate the optical absorption properties of these metal complexes. The geometry of **{3·2Co}$_3$** was optimized by GFN2-xTB semiempirical molecular orbital method and relative transfer free-energies for **{3·2Co}$_3$** and **3·2Co** computed with GFN2-xTB method.[10]

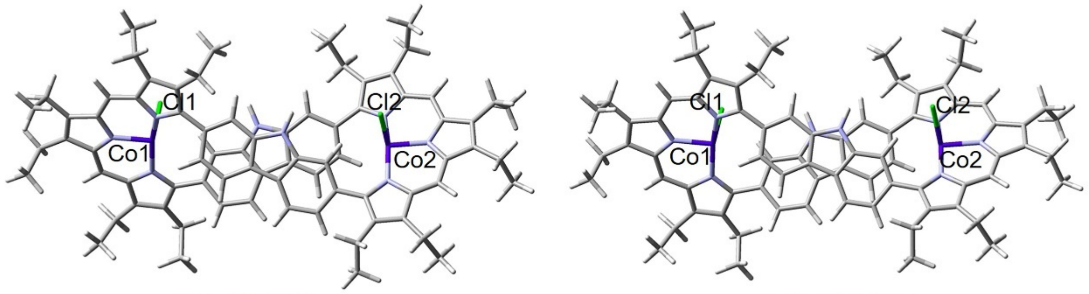

3·2Co (Singlet)
ΔE = 57.8 kcal mol^{-1}

3·2Co (Triplet)
ΔE = 0 kcal mol^{-1}

**3·2Co**	**Comp.**		**Exp.**
	**Singlet**	**Triplet**	
$d_{\text{Co1-N}}$	1.934	1.959	2.007
$d_{\text{Co2-N}}$	1.934	1.972	2.005
$d_{\text{Co1-Cl1}}$	2.338	2.510	2.324
$d_{\text{Co2-Cl2}}$	2.338	2.498	2.291
$d_{\text{Co1-Co2}}$	11.345	11.276	11.340

Figure S37. Relative energies and selected bond lengths (Å) involving the Co metal centres for the singlet and the triplet states obtained from the optimized structure ((U)B3LYP/6-311G(d,p)) and single crystal X-ray structure of **3·2Co**.

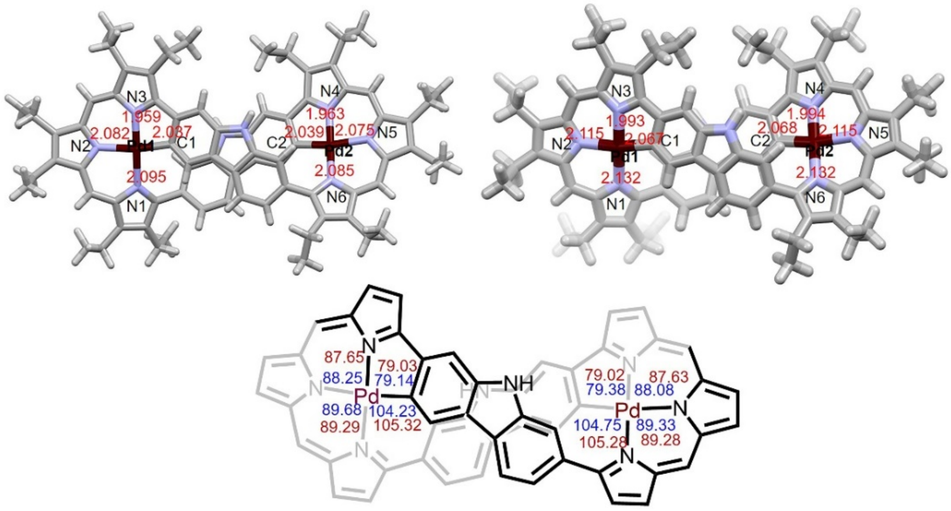

Figure S38. Comparison of selected bond lengths (numbers in Å in red) and bond angles (numbers in degrees blue for experiments and numbers in dark red for calculations) involving the structural parameters of **3·2Pd** around the Pd metal centres. Top view obtained from the single crystal X-ray structural data (left); top view obtained from DFT optimization (right) at the RB3LYP/6-311G(d,p)/LANL2DZ level.

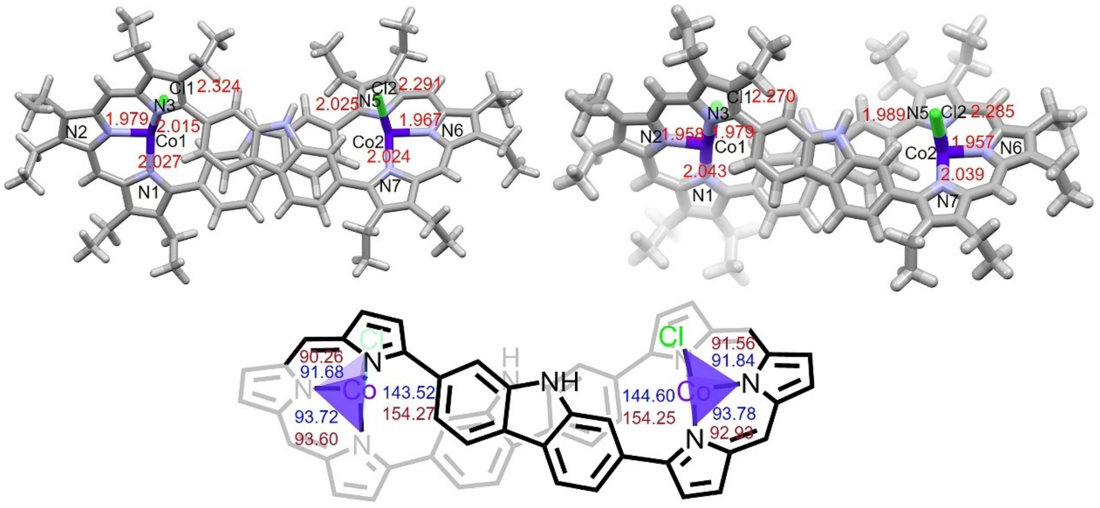

Figure S39. Comparison of selected bond length (numbers in Å in red) and bond angle (numbers in degrees in blue for experiments and numbers in dark red for calculations) involving the structural parameters of **3·2Co** around the Co metal centres. Top view obtained from the single crystal X-ray structural data (left); top view obtained from DFT centres (right) at the UB3LYP/6-311G(d,p)/LANL2DZ level.

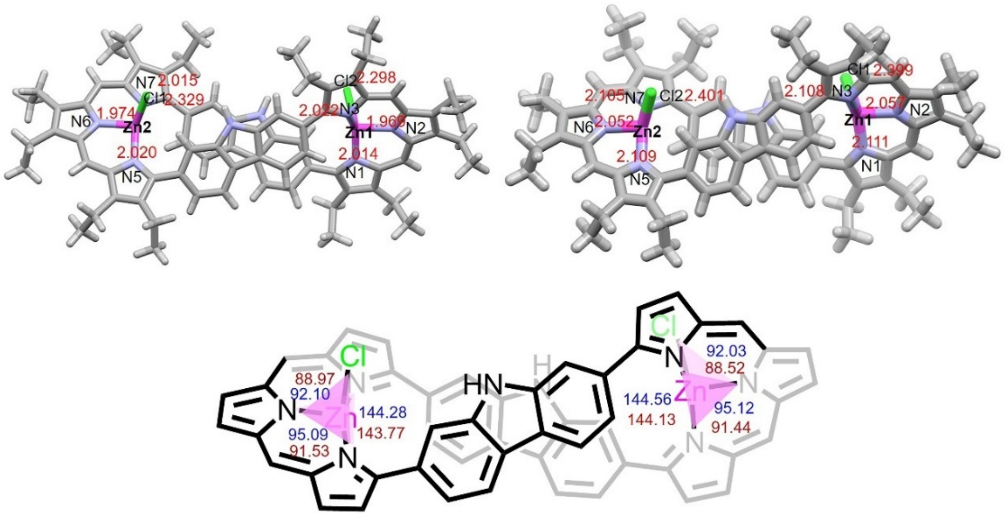

Figure S40. Comparison of selected bond length (numbers in Å in red) and bond angle (numbers in degrees in blue for experiments and numbers in dark red for calculations) involving the structural parameters of **3·2Zn** around the Zn metal centres. Top view obtained from the single crystal X-ray structural data (left); top view obtained from DFT optimization (right) at the RB3LYP/6-311G(d,p)/LANL2DZ level.

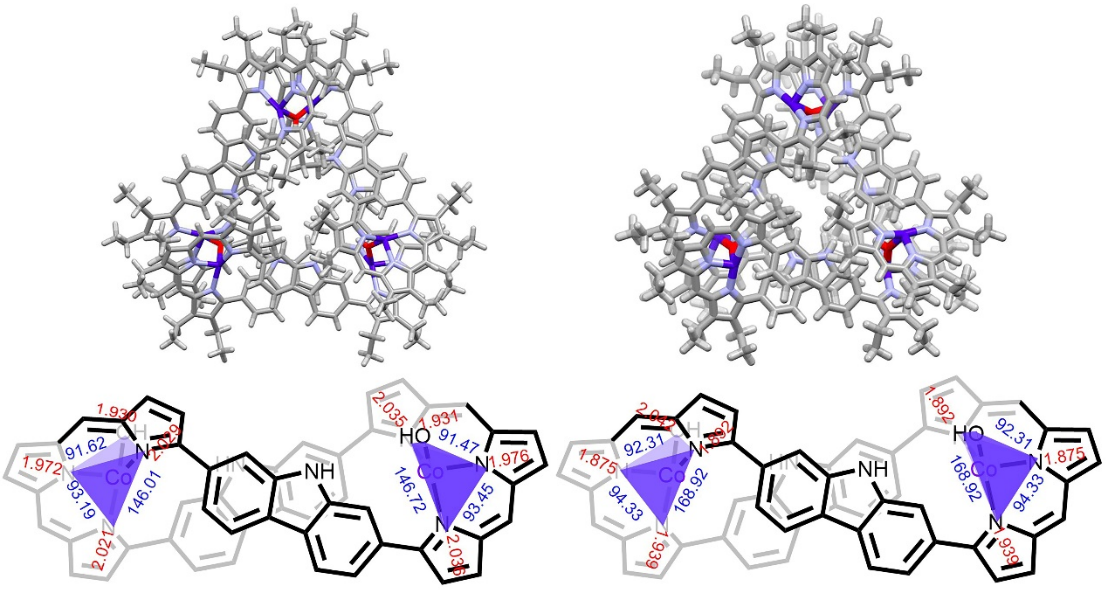

Figure S41. Comparison of selected bond length (numbers in Å in red) and bond angle (numbers in degrees in blue) involving the structural parameters of **{3·2Co}**$_3$ around the Co metal centres. Top view obtained from the single crystal X-ray structural data (left), top view obtained from semiempirical method (right) at the GFN2-xTB level.

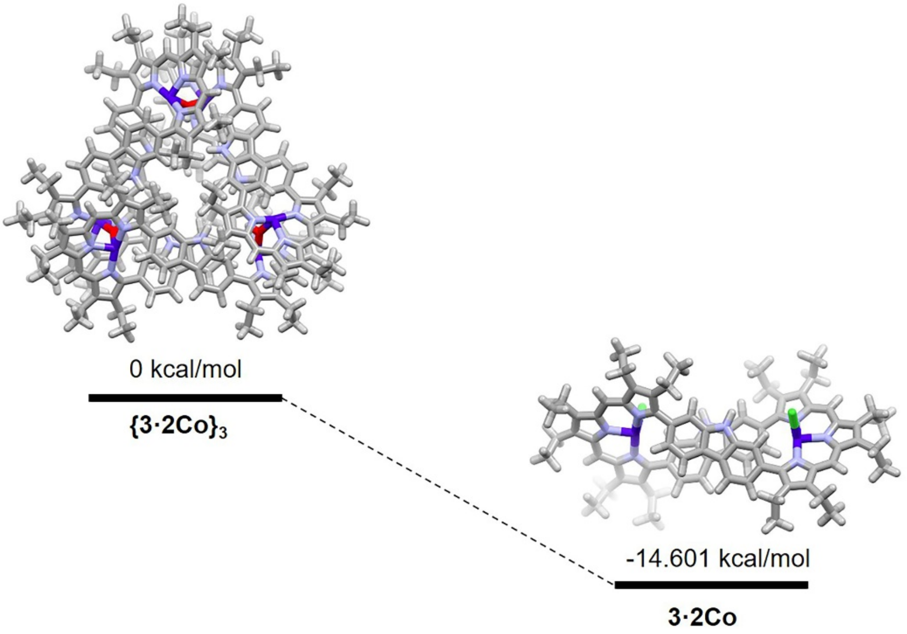

Figure S42. Gibbs free energy change at 298 K for the formation of metalloring **3·2Co** from the metallocage **{3·2Co}₃**.

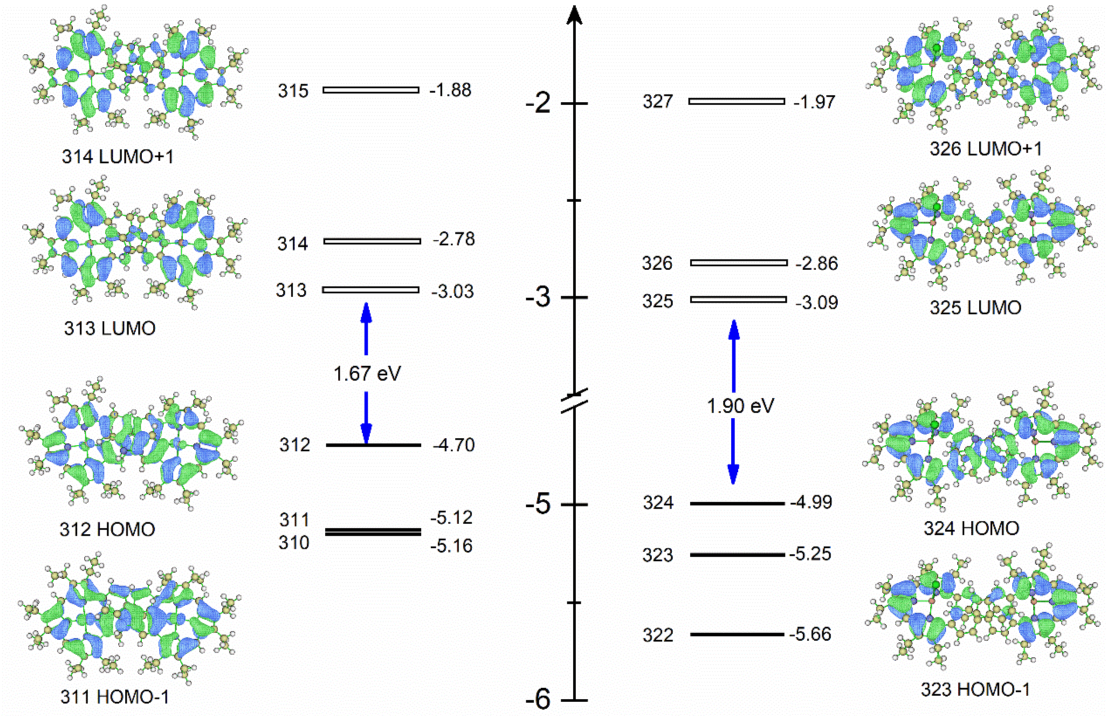

Figure S43. Comparison with the MO energy diagrams and selected frontier MOs for **3·2Pd** and **3·2Zn** calculated at the B3LYP/6-311G(d,p)/LANL2DZ level.

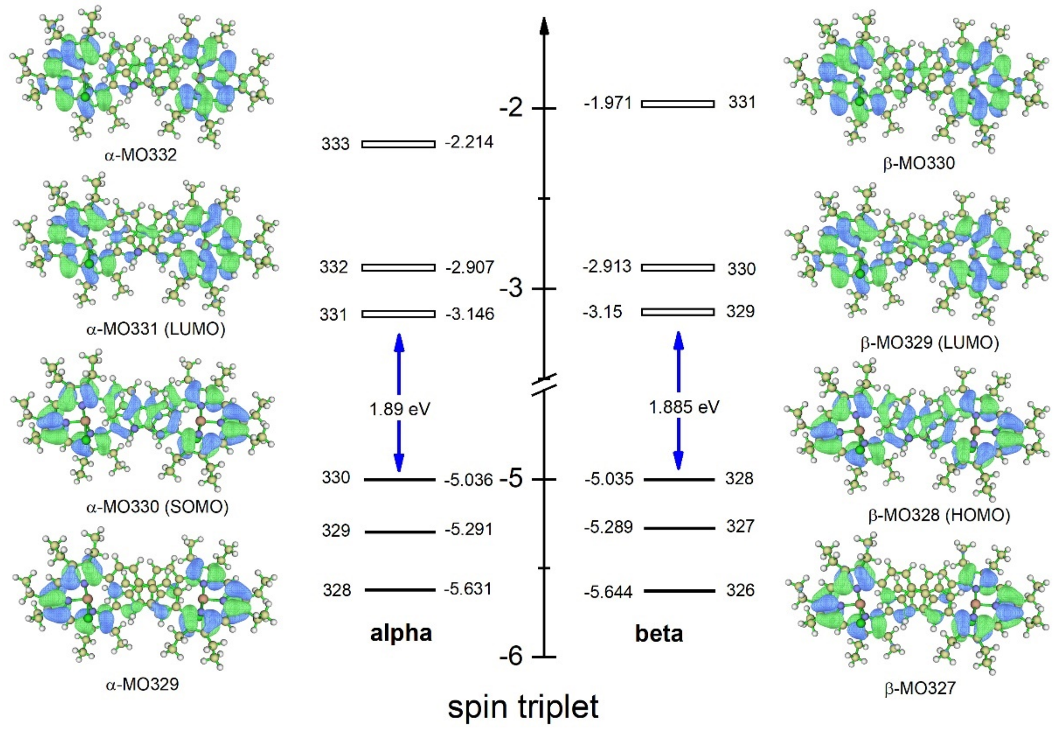

Figure S44. MO energy diagrams and selected frontier MOs for **3·2Co** calculated at the UB3LYP/6-311G(d,p)/ LANL2DZ level.

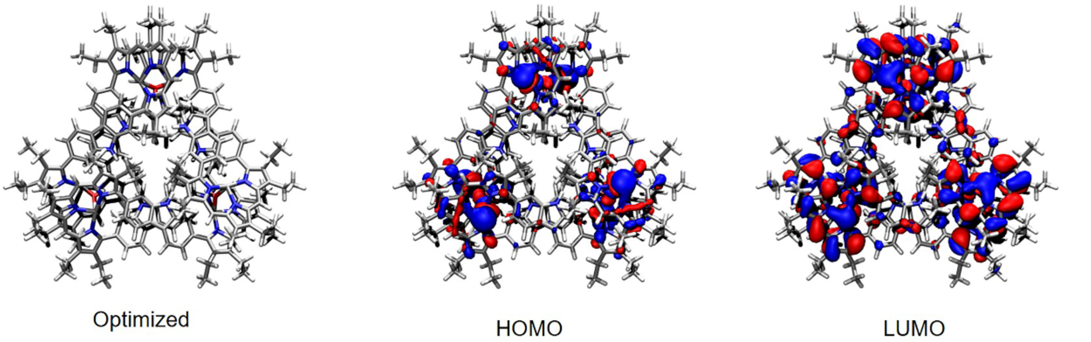


```
Note: Orbital   978 is HOMO, energy:   -0.326034 a.u.    -8.871847 eV
      Orbital   979 is LUMO, energy:   -0.280683 a.u.    -7.637763 eV
      HOMO-LUMO gap:    0.045352 a.u.     1.234084 eV    119.071007 kJ/mol
```

Figure S45. Optimized structure and selected frontier MOs for **{3·2Co}**$_3$ calculated by semiempirical molecular orbital methods with GFN2-xTB basic set.

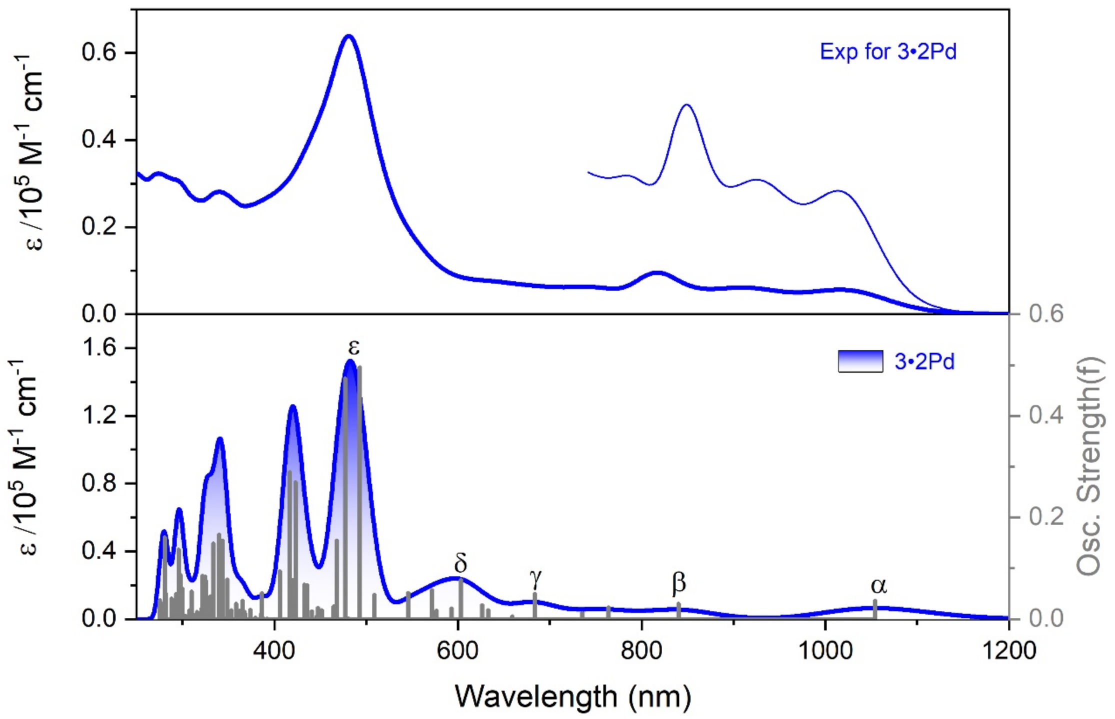

Figure S46. Comparison between the steady-state absorption (top) and TDDFT-predicted (bottom) spectra of **3·2Pd** calculated at the PBE0/Def2SVP level.

Table S3. Selected TD-DFT calculated excitation state energies, oscillator strengths and compositions of the major electronic transitions of **3·2Pd** calculated at the PBE0/Def2SVP level.

Peak	Sn	Wavelength [nm]	Oscillator Strength (f)	Energies [eV]	Major contributions
α	1	1054.29	0.0361	1.176	H→L (95.7%)
β	2	840.00	0.0298	1.476	H-1→L (52.4%), H→L+1 (38.8%)
γ	5	683.49	0.0493	1.814	H-2→L (78.2%)
δ	9	603.33	0.07701	2.055	H-3→L+1 (42.0%), H-6→L (23.1%) H-2→L (13.2%)
ε	15	492.98	0.4951	2.515	H→L+2 (51.3%), H-10→L (20.4%)
	17	477.05	0.4728	2.599	H-10→L (23.6%), H→L+2 (17.8%) H-5→L+1 (15.0%), H-6→L (12.3%)

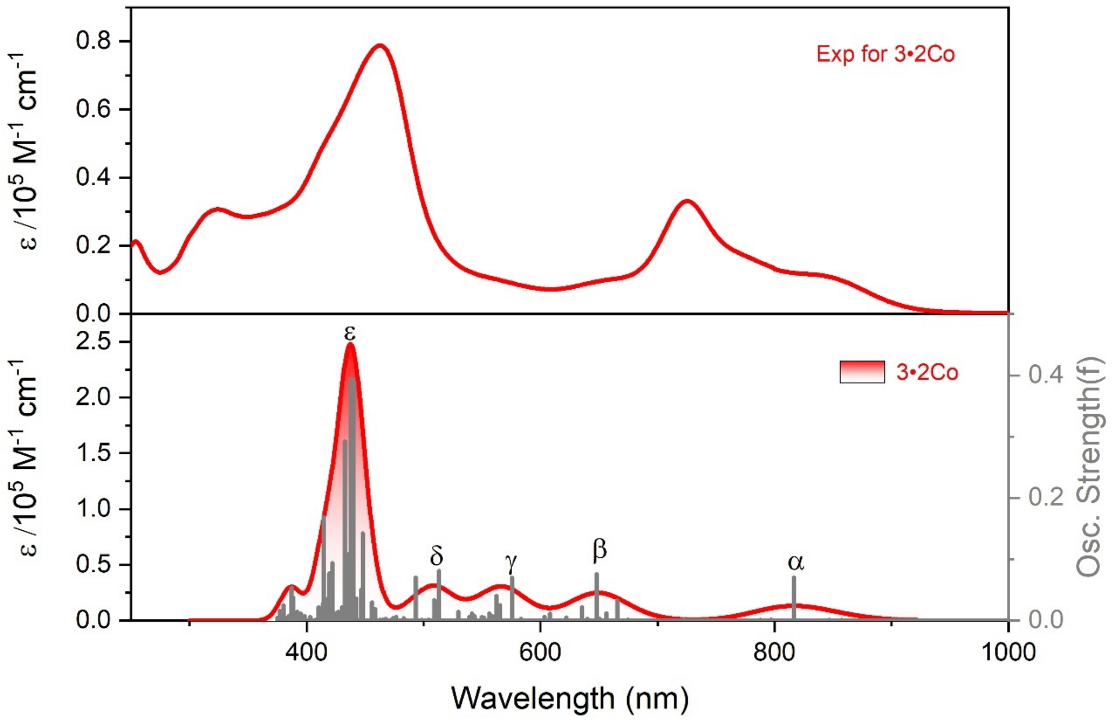

Figure S47. Comparison between the steady-state absorption (top) and TDDFT-predicted (bottom) spectra of **3·2Co** calculated at the PBE0/Def2SVP level.

Table S4. Selected TD-DFT calculated excitation state energies, oscillator strengths and compositions of the major electronic transitions of **3·2Co** at the PBE0/Def2SVP level.

Peak	Sn	Wavelength [nm]	Oscillator Strength (f)	Energies [eV]	Major contributions
α	15	816.76	0.0701	1.518	Hα→Lα (47.2%), Hβ→Lβ (46.5%)
β	25	647.78	0.0756	1.914	Hα-1→Lα (19.0%), Hβ→Lβ+1 (15.4%)
γ	34	575.60	0.0493	2.154	Hβ-2→Lβ (30.6%), Hα-2→Lα (26.2%)
	36	565.62	0.0248	2.192	Hα-1→Lα+1 (16.7%), Hβ-1→Lβ+1 (13.6%)
	37	562.03	0.0398	2.206	Hα-1→Lα+1 (23.1%), Hβ-1→Lβ+1 (16.6%)
δ	48	513.18	0.0806	2.416	Hβ-7→Lβ (15.5%)
	49	511.28	0.0213	2.425	Hβ-8→Lβ (15.3%), Hα-3→Lα+1(14.5%), Hα-2→Lα+1(13.5%)
	50	508.97	0.0334	2.436	Hβ-2→Lβ+1 (27.8%), Hα-2→Lα+1(23.4%), Hβ-8→Lβ (11.7%)
ε	67	440.28	0.3928	2.816	Hβ→Lβ+2 (15.6%), Hα-13→Lα (11.1%)
	68	437.64	0.3936	2.833	Hα-5→Lα+1 (11.8%), Hα-6→Lα (10.2%)
	69	436.41	0.1090	2.841	Hβ-8→Lβ+1 (25.1%), Hβ-6→Lβ+1 (10.6%)

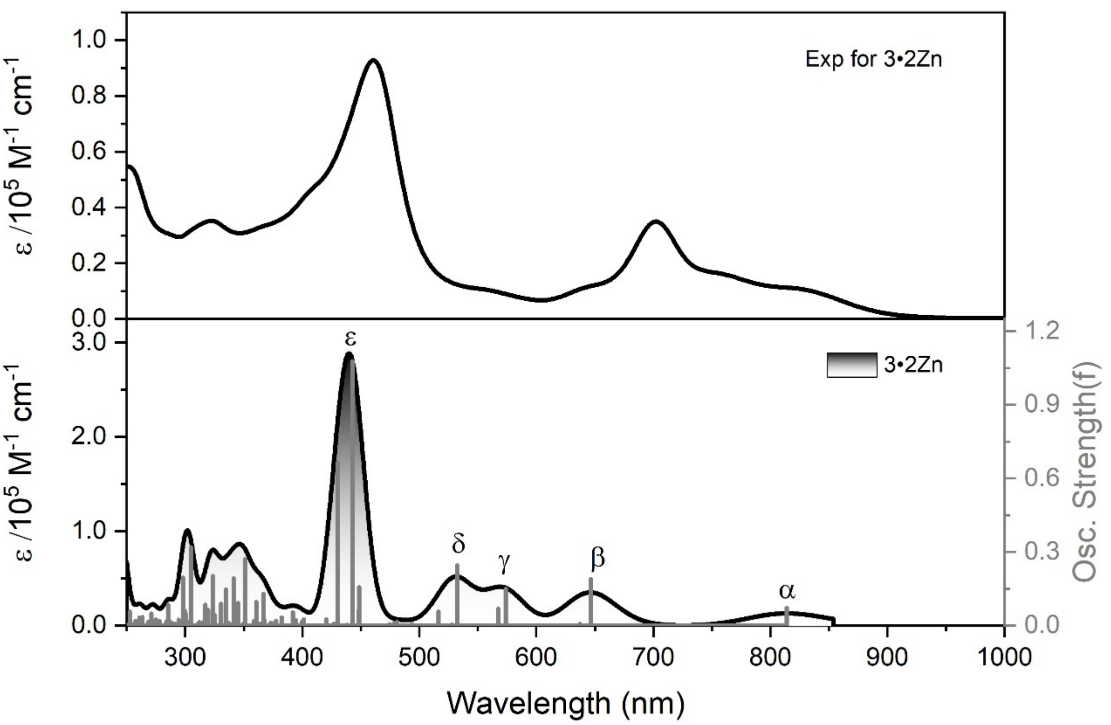

Figure S48. Comparison between the steady-state absorption (top) and TDDFT-predicted (bottom) spectra of **3·2Zn** calculated at the PBE0/Def2SVP level.

Table S5. Selected TD-DFT calculated excitation state energies, oscillator strengths and compositions of the major electronic transitions of **3·2Zn** at the PBE0/Def2SVP level.

Peak	Sn	Wavelength [nm]	Oscillator Strength (f)	Energies [eV]	Major contributions
α	1	814.08	0.0720	1.523	H→L (96.7%)
β	2	646.42	0.1891	1.918	H→L+1 (66.7%), H-1→L (25.2%)
γ	4	574.00	0.1500	2.160	H-2→L (84.0%), H-1→L (12.9%)
δ	6	532.35	0.2453	2.329	H-3→L (87.0%),
ε	15	442.64	1.0770	2.801	H→L+2 (61.8%), H-12→L (13.1%)
	19	430.20	0.6641	2.882	H-12→L (52.5%), H-13→L+1 (12.0%), H→L+2 (10.5%)

9. Supporting References

1. G. A. Bain, J. F. Berry, *J. Chem. Ed.* 2008, **85**, 532.
2. a) G. M. Sheldrick, *Methods. Enzymol.* 1997, **277**, 319; b) A. Altomare, M. C. Burla, M. Camalli, G. L. Cascarano, C. Giacovazzo, A. Guagliardi, A. G. G. Moliterni, G. Polidori, R. Spagna, *J. Appl. Cryst.* 1999, **32***,* 115−119.
3. W. N. Zhou. M. D. Hao, T. Lu, Z. M. Duan, T. Sarma, J. L. Sessler, C. H. Lei, *Chem.-Eur. J.* DOI: 10.1002/chem.202102939.
4. N. F. Chilton, R. P. Anderson, L. D. Turner, A. Soncini, K. S. Murray, *J. Chem. Comput.* 2013, **34**, 1164.
5. M. J. F. Gaussian 16 Revision A.03, G. W. Trucks, H. B. Schlegel, G. E. Scuseria, M. A. Robb, J. R. Cheeseman, G. Scalmani, V. Barone, G. A. Petersson, H. Nakatsuji, X. Li, M. Caricato, A. V. Marenich, J. Bloino, B. G. Janesko, R. Gomperts, B. Mennucci, H. P. Hratchian, J. V. Ortiz, A. F. Izmaylov, J. L. Sonnenberg, D. Williams, F. Ding, F. Lipparini, F. Egidi, J. Goings, B. Peng, A. Petrone, T. Henderson, D. Ranasinghe, V. G. Zakrzewski, J. Gao, N. Rega, G. Zheng, W. Liang, M. Hada, M. Ehara, K. Toyota, R. Fukuda, J. Hasegawa, M. Ishida, T. Nakajima, Y. Honda, O. Kitao, H. Nakai, T. Vreven, K. Throssell, J. A. Montgomery Jr., J. E. Peralta, F. Ogliaro, M. J. Bearpark, J. J. Heyd, E. N. Brothers, K. N. Kudin, V. N. Staroverov, T. A. Keith, R. Kobayashi, J. Normand, K. Raghavachari, A. P. Rendell, J. C. Burant, S. S. Iyengar, J. Tomasi, M. Cossi, J. M. Millam, M. Klene, C. Adamo, R. Cammi, J. W. Ochterski, R. L. Martin, K. Morokuma, O. Farkas, J. B. Foresman, and D. J. Fox, Gaussian Inc., Wallingford, CT, **2016**.
6. a) A. D. Becke, *J. Chem. Phys*. 1993, **98**, 1372; b) C. Lee, W. Yang, R. G. Parr, *Phys. Rev. B*. 1988, **37**, 785.
7. Tian Lu, Feiwu Chen, *Acta Chimica Sinica*. 2011, **69**, 2393-2406.
8. a) F. Neese, the ORCA Program System. *WIREs Comput. Mol. Sci*. 2012, **2**, 73-78; b) F. Neese, Software update: the ORCA program system, version 4.0. *WIREs Comput. Mol. Sci*. 2017, **8**, e1327.
9. a) J. P. Perdew, K. Burke, M. Ernzerhof, *Phys. Rev. Lett.* 1996, **77**, 3865-3868; b) F. Weigend, R. Ahlrichs, *Phys. Chem. Chem. Phys*. 2005, **7**, 3297-3305.
10. a) S. Grimme, C. Bannwarth, P. Shushkov, *J. Chem. Theory Comput.* 2017, **13**, 1989–2009; b) C. Bannwarth, S. Ehlert, S. Grimme, *J. Chem. Theory Comput.* 2019, **15**, 1652–1671.

Table S6: Cartesian coordinates of the S_0 optimized geometry of the **3·2Pd**.

* Sum of imaginary frequencies = 0

*Total Energy (hartree) = -3634.896191 Hartrees

Optimized structure Coordinates:

Atom	X	Y	Z
Pd	-5.06351	0.023084	0.329288
Pd	5.076701	-0.038695	-0.490826
N	-5.24723	-1.911389	-0.549587
N	-7.148687	0.294344	0.102335
N	-4.953935	1.945577	0.845914
N	0.917341	0.848727	1.880599
H	1.479283	1.683532	1.818991
N	5.254046	-1.895618	0.542042
N	7.163802	0.240561	-0.290782
N	4.97016	1.841811	-1.142194
N	-0.906046	0.688216	-2.07883
H	-1.466504	1.526264	-2.072133
C	-3.75329	2.476953	1.102941
C	-3.889162	3.914605	1.258648
C	-5.226556	4.201728	1.067709
C	-5.904588	2.944203	0.799142
C	-2.789504	4.895286	1.540213
H	-2.156811	4.518493	2.34959
H	-3.226951	5.825746	1.910004
C	-1.923713	5.204398	0.307891
H	-1.113805	5.892985	0.564515
H	-2.526411	5.664484	-0.479936
H	-1.477778	4.296211	-0.105045
C	-5.906705	5.532927	1.171747
H	-5.198349	6.326461	0.920444
H	-6.709573	5.591503	0.430499
C	-10.668696	-0.957033	-0.046699
H	-11.467519	-0.444931	0.496628
H	-10.551895	-1.933745	0.433342
C	-6.488406	5.794131	2.571207
H	-6.983064	6.768975	2.609863
H	-5.700735	5.780237	3.329004
H	-7.221	5.028872	2.840224
C	-7.238084	2.710936	0.552879
H	-7.902723	3.566039	0.587505
C	-7.836791	1.457516	0.294989
C	-9.255458	1.192091	0.260763
C	-9.396492	-0.172953	0.083699

C	-8.060251	-0.705488	-0.020262
C	-10.341504	2.207208	0.46146
H	-10.078765	3.137635	-0.052467
H	-11.261494	1.849364	-0.008537
C	-10.614575	2.508317	1.944214
H	-9.718812	2.894164	2.43798
H	-10.919515	1.602433	2.474969
H	-11.410458	3.251335	2.052165
C	-11.100132	-1.160751	-1.508137
H	-11.266634	-0.199818	-2.002448
H	-12.027091	-1.739198	-1.565641
H	-10.330923	-1.693775	-2.073341
C	-7.71145	-2.047765	-0.259635
H	-8.542823	-2.739853	-0.319155
C	-6.473865	-2.587434	-0.528457
C	-6.290798	-3.91623	-1.077366
C	-4.982207	-4.002182	-1.486623
C	-4.365772	-2.727787	-1.158536
C	-7.376611	-4.925339	-1.303123
H	-8.091489	-4.896474	-0.475594
H	-6.940724	-5.927127	-1.295068
C	-8.122905	-4.710395	-2.630961
H	-8.59678	-3.72622	-2.659724
H	-8.900021	-5.468202	-2.765752
H	-7.435944	-4.772224	-3.478819
C	-3.930354	-6.310307	-1.285068
H	-3.198911	-5.973992	-0.545705
H	-3.480731	-7.125389	-1.85967
H	-4.784984	-6.713807	-0.736026
C	-3.006244	-2.319588	-1.489539
C	-1.934349	-3.239997	-1.354364
H	-2.145358	-4.261608	-1.075906
C	-0.617631	-2.834351	-1.46698
H	0.186897	-3.540615	-1.30698
C	-0.337216	-1.484706	-1.707714
C	0.865486	-0.691073	-1.65076
C	2.197954	-0.979007	-1.301244
H	2.475469	-2.003907	-1.091839
C	3.113689	0.043922	-1.13292
C	2.6959	1.389398	-1.450547
C	1.373273	1.700633	-1.793953
H	1.061124	2.716474	-1.991174
C	0.464179	0.655416	-1.860371
C	-1.417868	-0.586847	-1.927168

C	-2.743211	-0.991281	-1.844289
H	-3.553986	-0.285546	-1.960414
C	3.768787	2.358683	-1.425934
C	3.907549	3.781479	-1.679349
C	5.251124	4.072452	-1.536389
C	5.926118	2.834614	-1.183262
C	2.804982	4.74944	-1.989222
H	2.158322	4.33932	-2.770988
H	3.23953	5.662571	-2.403993
C	1.956212	5.116215	-0.760401
H	2.570173	5.605206	0.000507
H	1.509149	4.229304	-0.304589
H	1.147753	5.798582	-1.037213
C	5.922157	5.40637	-1.653166
H	5.394033	6.017065	-2.390546
H	6.938152	5.270502	-2.035509
C	5.982608	6.165429	-0.317187
H	6.491787	7.125758	-0.439881
H	6.522258	5.587075	0.437354
H	4.977733	6.359313	0.06643
C	7.262821	2.610935	-0.941266
H	7.933412	3.452116	-1.070798
C	7.856755	1.381444	-0.580866
C	9.274516	1.11146	-0.531622
C	9.409607	-0.232568	-0.231978
C	10.365396	2.12195	-0.730582
H	11.282469	1.609237	-1.032331
H	10.105604	2.791184	-1.556773
C	10.643798	2.957276	0.529562
H	11.443701	3.681727	0.349571
H	10.945783	2.316	1.362076
H	9.751868	3.506723	0.842692
C	10.675893	-1.028536	-0.116533
H	11.505777	-0.35579	0.115191
H	10.602177	-1.722113	0.727598
C	11.006237	-1.819587	-1.392613
H	11.126528	-1.146792	-2.246069
H	10.20591	-2.52313	-1.637188
H	11.933776	-2.387271	-1.271954
C	8.070455	-0.749654	-0.088404
C	7.716816	-2.065311	0.26873
H	8.546928	-2.749322	0.397782
C	6.478609	-2.575238	0.584355
C	6.292688	-3.841782	1.267367

C	4.98807	-3.875317	1.69688
C	4.374413	-2.638834	1.236224
C	7.379588	-4.83119	1.562579
H	8.110925	-4.831056	0.749615
H	6.95291	-5.837485	1.584981
C	8.096262	-4.55683	2.894904
H	8.545222	-3.560227	2.900256
H	8.889405	-5.290257	3.06662
H	7.396225	-4.610032	3.732273
C	4.387669	-4.903786	2.614369
H	3.469147	-5.320296	2.194323
H	5.085139	-5.741018	2.694748
C	4.094062	-4.362081	4.022147
H	3.362424	-3.552253	3.991792
H	5.004848	-3.973734	4.485944
H	3.695161	-5.153366	4.66347
C	3.011692	-2.198368	1.512651
C	1.939155	-3.122664	1.428031
H	2.151732	-4.161237	1.219795
C	0.623328	-2.706826	1.503824
H	-0.182704	-3.419149	1.383966
C	0.345832	-1.343358	1.654057
C	-0.855383	-0.553316	1.5416
C	-2.186941	-0.86313	1.2089
H	-2.464274	-1.899727	1.067491
C	-3.101736	0.14667	0.970375
C	-2.68246	1.510091	1.19741
C	-1.359966	1.843116	1.521327
H	-1.046937	2.869945	1.647459
C	-0.452553	0.803647	1.661127
C	1.427179	-0.434701	1.816574
C	2.752239	-0.847762	1.769064
H	3.565391	-0.138982	1.842737
C	-4.364956	-5.164707	-2.21332
H	-3.51116	-4.823487	-2.801903
H	-5.091768	-5.556467	-2.931454

Table S7: Cartesian coordinates of the S_0 optimized geometry of the **3·2Co**.

* Sum of imaginary frequencies = 0

*Total Energy (hartree) = -4593.409978 Hartrees

Optimized structure Coordinates:

Atom	X	Y	Z
Co	-5.641626	-0.108194	0.046184
Co	5.633571	-0.120291	-0.059578
Cl	-5.400457	-1.913738	1.77383
Cl	5.403481	-1.898141	-1.799848
N	-5.80728	1.379979	1.324537
N	-7.574837	-0.272258	-0.19986
N	-5.293131	-1.349086	-1.417373
N	-0.815177	-0.815433	1.669967
H	-1.216491	-1.740079	1.712974
N	5.286263	-1.364665	1.419998
N	7.577648	-0.277306	0.192771
N	5.796446	1.406053	-1.316391
N	0.80763	-0.779321	-1.665766
H	1.208853	-1.703835	-1.712173
C	-4.088579	-1.652778	-1.928046
C	-4.183324	-2.817101	-2.793013
C	-5.509862	-3.192169	-2.792143
C	-6.205229	-2.262844	-1.933022
C	-3.086501	-3.453853	-3.59643
H	-2.304201	-2.720253	-3.801579
H	-3.491287	-3.749661	-4.569042
C	-2.472799	-4.69339	-2.926547
H	-3.22366	-5.477132	-2.80065
H	-2.076194	-4.459258	-1.934878
H	-1.656997	-5.097304	-3.53307
C	-6.146688	-4.333994	-3.521493
H	-7.097666	-4.008562	-3.955139
H	-5.507724	-4.631238	-4.356949
C	-6.399018	-5.551588	-2.615643
H	-6.85534	-6.366336	-3.185021
H	-7.067556	-5.294494	-1.790404
H	-5.463574	-5.916669	-2.184872
C	-7.556503	-2.203509	-1.701196
H	-8.174297	-2.951271	-2.182493
C	-8.215482	-1.248415	-0.911519
C	-9.638181	-1.159261	-0.703216
C	-9.840744	-0.10265	0.16559
C	-8.536088	0.422691	0.475727

C	-10.671024	-2.025942	-1.358396
H	-10.31975	-3.062024	-1.389916
H	-11.57724	-2.026981	-0.746977
C	-11.023597	-1.568291	-2.783127
H	-11.780169	-2.223034	-3.225405
H	-10.14164	-1.582732	-3.429317
H	-11.416491	-0.548135	-2.777833
C	-11.14248	0.438881	0.675317
H	-11.034628	0.738666	1.722142
H	-11.892976	-0.356056	0.659615
C	-11.656984	1.634729	-0.142311
H	-12.60514	2.002128	0.261121
H	-11.817659	1.351887	-1.185994
H	-10.939111	2.459294	-0.12706
C	-8.260413	1.460905	1.376831
H	-9.115905	1.951458	1.824001
C	-7.021992	1.912199	1.76001
C	-6.768153	3.014739	2.65661
C	-5.400787	3.156328	2.733669
C	-4.83364	2.11867	1.889467
C	-7.817175	3.832188	3.345758
H	-7.367601	4.336401	4.204883
H	-8.594551	3.171933	3.743132
C	-8.470898	4.880187	2.428465
H	-8.976626	4.403757	1.584999
H	-7.723636	5.567866	2.025149
H	-9.210586	5.465821	2.98163
C	-4.663212	4.132478	3.606805
H	-3.644402	3.780045	3.775169
H	-5.146672	4.150242	4.589073
C	-4.630484	5.566503	3.056517
H	-5.639754	5.976655	2.97631
H	-4.17984	5.605747	2.061078
H	-4.051258	6.219115	3.716233
C	-3.403316	1.860488	1.722599
C	-2.502347	2.950544	1.613668
H	-2.894043	3.955896	1.559874
C	-1.132825	2.755415	1.558175
H	-0.461823	3.602711	1.48045
C	-0.629345	1.451993	1.607425
C	0.696545	0.886803	1.616133
C	1.984953	1.426394	1.616396
H	2.135099	2.498047	1.58678
C	3.071157	0.570767	1.676659

H	4.073285	0.979596	1.74652
C	2.909254	-0.838538	1.735266
C	1.621419	-1.39168	1.70499
H	1.470699	-2.461028	1.710968
C	0.535124	-0.528007	1.659438
C	-1.539554	0.362317	1.660005
C	-2.914868	0.548937	1.724017
H	-3.58777	-0.293167	1.830479
C	4.085319	-1.683095	1.930096
C	4.189662	-2.857199	2.779002
C	5.519448	-3.223446	2.769948
C	6.207142	-2.278583	1.921381
C	3.098338	-3.512471	3.574996
H	2.31358	-2.785668	3.794766
H	3.507317	-3.822864	4.541258
C	2.487442	-4.74312	2.886419
H	2.086135	-4.49388	1.900356
H	1.675632	-5.160767	3.488959
H	3.241118	-5.521386	2.744465
C	6.163443	-4.373114	3.480559
H	5.529063	-4.684127	4.31451
H	7.115291	-4.050904	3.914617
C	6.415423	-5.577224	2.556646
H	5.479198	-5.939423	2.125183
H	6.876924	-6.398271	3.112632
H	7.079367	-5.306601	1.732053
C	7.558353	-2.214654	1.6837
H	8.177051	-2.966183	2.158178
C	8.219058	-1.255606	0.898622
C	9.641908	-1.162752	0.689846
C	9.841773	-0.099262	-0.171966
C	8.535207	0.426132	-0.476946
C	10.677136	-2.032173	1.337504
H	10.32833	-3.069383	1.359816
H	11.583524	-2.025438	0.726345
C	11.028212	-1.586644	2.766455
H	10.146164	-1.609365	3.41228
H	11.418391	-0.565451	2.770646
H	11.786398	-2.243464	3.202867
C	11.142499	0.447722	-0.678256
H	11.892121	-0.348278	-0.676906
H	11.031246	0.763085	-1.720111
C	11.661357	1.630787	0.155104
H	10.943986	2.455956	0.155067

H	12.608192	2.003498	-0.246577
H	11.825994	1.332129	1.193749
C	8.253742	1.474889	-1.36599
H	9.108837	1.969942	-1.809359
C	7.015823	1.935834	-1.743255
C	6.768773	3.049471	-2.628941
C	5.401795	3.197955	-2.708702
C	4.828181	2.153987	-1.87736
C	7.822505	3.870945	-3.306039
H	7.377388	4.385833	-4.161151
H	8.599205	3.212659	-3.707949
C	8.476433	4.906998	-2.375327
H	7.730116	5.593081	-1.967601
H	9.220222	5.495761	-2.919593
H	8.977297	4.420053	-1.534943
C	4.670899	4.187153	-3.572675
H	5.158317	4.215813	-4.5527
H	3.651728	3.83973	-3.749407
C	4.639733	5.614405	-3.004879
H	4.066109	6.276894	-3.659627
H	5.649786	6.020413	-2.914193
H	4.183761	5.642921	-2.011528
C	3.396576	1.89654	-1.713933
C	2.495716	2.985886	-1.597952
H	2.887538	3.990897	-1.539111
C	1.125902	2.790771	-1.540831
H	0.455655	3.638191	-1.457931
C	0.621327	1.487937	-1.595526
C	-0.705009	0.922309	-1.603458
C	-1.994396	1.460227	-1.600563
H	-2.145786	2.531587	-1.568416
C	-3.080362	0.603612	-1.662113
H	-4.083113	1.011921	-1.731525
C	-2.916483	-0.805299	-1.724714
C	-1.627906	-1.356708	-1.696747
H	-1.475791	-2.42582	-1.70612
C	-0.542585	-0.492281	-1.65098
C	1.531741	0.398442	-1.654242
C	2.906882	0.585026	-1.720912
H	3.57898	-0.257102	-1.832514

Table S8: Cartesian coordinates of the S_0 optimized geometry of the **3·2Zn**.

* Sum of imaginary frequencies = 0

*Total Energy (hartree) = -4434.469029 Hartrees

Optimized structure Coordinates:

Atom	X	Y	Z
Zn	5.584768	-0.434423	0.432159
Zn	-5.572036	-0.469293	-0.491648
Cl	5.017106	-2.214232	1.940324
Cl	-5.028662	-2.187338	-2.075883
N	5.720783	1.431655	1.405303
N	7.594863	-0.427643	0.021193
N	5.246361	-1.358127	-1.428712
N	-0.808973	-0.704749	-1.883022
H	-1.224937	-1.6135	-2.019814
N	-5.722734	1.422192	-1.418981
N	-7.578607	-0.467302	-0.042964
N	-5.219158	-1.449663	1.340109
N	0.836951	-0.756441	1.760152
H	1.264859	-1.66334	1.870023
C	4.787981	2.255053	1.910473
C	5.401105	3.340414	2.649676
C	6.76256	3.129275	2.585261
C	6.970029	1.934857	1.795126
C	4.706231	4.430837	3.414675
H	5.218543	4.566976	4.372519
H	3.688987	4.11715	3.656588
C	4.669918	5.782469	2.685186
H	4.113216	6.522114	3.268024
H	4.194689	5.696541	1.704197
H	5.67963	6.168064	2.526486
C	7.843431	3.97309	3.188824
H	8.609792	3.329949	3.632667
H	7.421054	4.560388	4.008361
C	8.507911	4.922483	2.176967
H	7.77258	5.596341	1.731039
H	8.980635	4.364405	1.365266
H	9.275593	5.529488	2.665223
C	8.211229	1.413556	1.479739
H	9.054713	1.940983	1.910492
C	8.537757	0.319895	0.652973
C	9.854183	-0.146458	0.288976
C	9.662999	-1.202766	-0.590212
C	8.235773	-1.346077	-0.746418

C	11.152755	0.453074	0.737692
H	11.060693	0.797051	1.772508
H	11.926259	-0.320357	0.739875
C	11.614318	1.624607	-0.144593
H	10.870781	2.426017	-0.154655
H	11.76411	1.299691	-1.177295
H	12.557607	2.040215	0.22196
C	10.706111	-2.004985	-1.308219
H	11.636646	-1.982228	-0.73427
H	10.396761	-3.054372	-1.351056
C	10.980476	-1.503472	-2.735573
H	10.07273	-1.535357	-3.344174
H	11.740364	-2.119609	-3.225269
H	11.335765	-0.469982	-2.724147
C	7.550021	-2.23527	-1.606015
H	8.163515	-2.967182	-2.118765
C	6.20732	-2.249078	-1.920699
C	5.573989	-3.126503	-2.885703
C	4.251134	-2.747482	-2.966774
C	4.093996	-1.635035	-2.044907
C	6.257535	-4.2294	-3.632882
H	7.239365	-3.8886	-3.976829
H	5.680827	-4.469668	-4.529962
C	6.435648	-5.502585	-2.787864
H	5.469546	-5.882982	-2.447827
H	7.043028	-5.304101	-1.901529
H	6.926595	-6.286569	-3.371488
C	3.211398	-3.325407	-3.882554
H	3.677675	-3.559068	-4.844755
H	2.446985	-2.573366	-4.089933
C	2.549054	-4.600391	-3.337437
H	2.091835	-4.429123	-2.359078
H	3.284783	-5.399029	-3.216396
H	1.771036	-4.955476	-4.019475
C	2.915716	-0.789568	-1.852395
C	3.100592	0.60529	-1.669946
H	4.109014	0.998368	-1.660565
C	2.026149	1.472792	-1.579153
H	2.193099	2.535386	-1.45837
C	0.728208	0.961179	-1.658414
C	-0.589115	1.549369	-1.64504
C	-1.081078	2.852538	-1.519771
H	-0.402452	3.683534	-1.368083
C	-2.447887	3.072646	-1.58756

H	-2.825833	4.076817	-1.461199
C	-3.361312	2.00794	-1.79186
C	-2.880428	0.697053	-1.898763
H	-3.544369	-0.129528	-2.11194
C	-1.512603	0.482173	-1.806915
C	0.546144	-0.442505	-1.816078
C	1.619962	-1.319567	-1.908103
H	1.454603	-2.382236	-2.012436
C	-4.799397	2.266382	-1.906465
C	-5.425951	3.376913	-2.596274
C	-6.785664	3.159839	-2.519748
C	-6.978763	1.937321	-1.769973
C	-4.745318	4.497649	-3.329968
H	-5.269872	4.665351	-4.276097
H	-3.729599	4.198156	-3.595089
C	-4.70698	5.82282	-2.553389
H	-5.716759	6.194988	-2.365761
H	-4.16451	6.586782	-3.11799
H	-4.215906	5.706337	-1.583363
C	-7.877765	4.02248	-3.074724
H	-8.647548	3.394354	-3.534066
H	-7.468602	4.643886	-3.875567
C	-8.532357	4.928057	-2.017168
H	-8.994131	4.336188	-1.223196
H	-9.307157	5.552359	-2.471156
H	-7.793252	5.585202	-1.552893
C	-8.214568	1.405101	-1.452403
H	-9.065032	1.943318	-1.855292
C	-8.529195	0.286589	-0.654772
C	-9.840428	-0.210205	-0.314048
C	-9.638599	-1.294877	0.527253
C	-8.21009	-1.424441	0.685213
C	-11.144731	0.382896	-0.754224
H	-11.904621	-0.403103	-0.792186
H	-11.048333	0.764668	-1.775283
C	-11.635493	1.514268	0.163922
H	-12.580883	1.928169	-0.199129
H	-10.904901	2.326172	0.212188
H	-11.792551	1.149574	1.182152
C	-10.675079	-2.136963	1.208096
H	-11.599917	-2.110794	0.625094
H	-10.34911	-3.18204	1.220679
C	-10.972976	-1.685595	2.647359
H	-11.727021	-2.329788	3.109168

H	-11.345626	-0.658334	2.664575
H	-10.071383	-1.721138	3.264635
C	-7.514713	-2.346417	1.501319
H	-8.119347	-3.110274	1.976315
C	-6.169407	-2.364726	1.806208
C	-5.516458	-3.284979	2.716131
C	-4.192675	-2.908956	2.788272
C	-4.053606	-1.753335	1.919574
C	-6.194187	-4.365596	3.50302
H	-6.968292	-4.839093	2.892004
H	-5.469144	-5.148063	3.739398
C	-6.821457	-3.844268	4.80704
H	-7.572583	-3.077323	4.6024
H	-6.061454	-3.399825	5.454974
H	-7.304586	-4.657418	5.35616
C	-3.136731	-3.551087	3.641236
H	-3.589011	-3.875659	4.582813
H	-2.376254	-2.813208	3.90706
C	-2.471739	-4.764419	2.971397
H	-3.207975	-5.545693	2.7656
H	-1.695992	-5.188275	3.615659
H	-2.01185	-4.494772	2.016987
C	-2.885611	-0.890071	1.746283
C	-3.089122	0.507949	1.609023
H	-4.102337	0.888301	1.620631
C	-2.027135	1.392301	1.539224
H	-2.209259	2.455879	1.454418
C	-0.722005	0.896145	1.595591
C	0.587503	1.501734	1.599131
C	1.063756	2.814708	1.522206
H	0.37509	3.642342	1.400319
C	2.427657	3.049787	1.602381
H	2.792659	4.063031	1.515638
C	3.353709	1.989788	1.771503
C	2.888718	0.670347	1.826262
H	3.558821	-0.155639	2.020189
C	1.524431	0.44146	1.723455
C	-0.521412	-0.509553	1.7071
C	-1.582549	-1.403649	1.777137
H	-1.400939	-2.466319	1.846319

Table S9: Cartesian coordinates of the S_0 optimized geometry of the {**3·2Co**}$_3$.
* Sum of imaginary frequencies = 0
*Total Energy (hartree) = -18658.316727546164 Hartrees

Optimized structure Coordinates:

Atom	X	Y	Z
Co	-0.73811351	10.94128293	5.69562279
Co	-3.23969408	11.68893807	2.74853767
O	-1.83422595	11.83367074	4.2223324
N	0.12586502	9.50645655	4.71847548
N	-2.12397298	9.85684145	6.34333498
N	-1.25180184	12.38922028	6.80045452
N	3.54084658	15.96208859	5.57648039
H	3.27173465	16.92943768	5.63430044
N	8.44595878	17.36570617	3.72533575
N	8.22425637	19.63198759	2.10066677
N	5.64604279	18.90579742	1.64425422
N	1.80602264	14.32455981	2.86941118
H	0.85560459	14.64862761	2.81281477
C	1.25312305	9.52660482	3.98467591
C	1.56851865	8.21602838	3.48137409
C	0.57774664	7.38273816	3.93172722
C	-0.31925212	8.20132049	4.70075861
C	2.69413244	7.81650929	2.58042402
H	3.0944589	8.69358791	2.06939247
H	2.30317711	7.14018413	1.81660345
C	3.81573963	7.08162379	3.32179042
H	4.65089735	6.90269223	2.6493318
H	3.46301936	6.12240701	3.68846451
H	4.17564414	7.65153619	4.17579779
C	0.44419903	5.91232381	3.74933701
H	1.0664169	5.58935732	2.91402182
H	-0.59247922	5.66255339	3.51651008
C	0.86934374	5.15266359	5.01127743
H	0.77457356	4.08206244	4.85101089
H	0.2505177	5.43409705	5.85961383
H	1.9036343	5.37308962	5.26201699
C	-1.46259986	7.75464969	5.31076263
H	-1.72752035	6.71766317	5.17734063
C	-2.32005352	8.5426234	6.07352281
C	-3.54516246	8.08433899	6.66926521
C	-4.12089252	9.17827561	7.25748809
C	-3.22711073	10.28056376	7.02675388
C	-4.00631966	6.66663032	6.6841604

H	-5.06549376	6.63060407	6.94152302
H	-3.88192786	6.22146546	5.69410908
C	-3.22342145	5.84155003	7.71022283
H	-2.15860313	5.85951011	7.49155477
H	-3.56427179	4.80980211	7.70088629
H	-3.370567	6.24281536	8.70930131
C	-5.35842543	9.24786187	8.08339186
H	-6.02654616	8.43077282	7.8092187
H	-5.87725787	10.18859016	7.89377262
C	-5.028536	9.14418961	9.5756829
H	-4.35467067	9.94334356	9.87556655
H	-4.54829517	8.19425035	9.79329753
H	-5.93741025	9.21319885	10.16741903
C	-3.43206215	11.57837811	7.46725963
H	-4.36882868	11.80954673	7.94968271
C	-2.48588039	12.57668777	7.37128449
C	-2.56300089	13.88855182	7.93425671
C	-1.33905279	14.4803371	7.70707751
C	-0.55271915	13.52344316	6.98622638
C	-3.74200877	14.43025358	8.66837974
H	-4.65939455	14.04803133	8.21506703
H	-3.76671238	15.51942221	8.58646361
C	-3.70380864	14.03960926	10.1484083
H	-2.82398661	14.45795073	10.63048668
H	-3.67105009	12.95846594	10.25699396
H	-4.58699518	14.41435936	10.65891063
C	-0.89404621	15.77390842	8.31124489
H	-1.76765564	16.27578294	8.72769101
H	-0.45412168	16.44794769	7.57346001
C	0.11482572	15.51759508	9.43213484
H	1.02012125	15.06144731	9.0371836
H	-0.31022941	14.84128688	10.16925369
H	0.37688804	16.44738502	9.93109021
C	0.83057521	13.63152627	6.5672731
C	1.61550991	12.46101955	6.54159187
H	1.15599148	11.51770064	6.85030176
C	2.95918837	12.49564829	6.23932941
H	3.55122115	11.59483272	6.25504556
C	3.54260244	13.7162578	5.92035719
C	4.85977757	14.11837519	5.53267513
C	6.03568162	13.42341934	5.27928499
H	6.07226401	12.35144108	5.39059167
C	7.14410864	14.12832345	4.87377601
H	8.03464523	13.5881675	4.60607831

C	7.13177865	15.5319272	4.75583329
C	5.94716704	16.23421165	4.97538293
H	5.94743998	17.31181958	4.91201851
C	4.82270903	15.52082572	5.34540306
C	2.75604842	14.89510533	5.93077888
C	1.41096399	14.85835627	6.23921421
H	0.82560099	15.76237838	6.20944575
C	8.36173088	16.24099824	4.4584626
C	9.65218654	15.85141303	4.96174238
C	10.54042535	16.7936685	4.51227228
C	9.77423306	17.73608816	3.74358533
C	9.98689275	14.70417106	5.86179705
H	10.68416609	15.05537764	6.62609151
H	9.08835028	14.35389414	6.37229643
C	10.6569424	13.54327203	5.11948787
H	10.0675891	13.21714427	4.26510806
H	10.78801133	12.69867634	5.7911886
H	11.63470672	13.84123098	4.7532122
C	12.01598088	16.84359891	4.69518928
H	12.32393014	17.86430102	4.92893017
H	12.30288997	16.20343128	5.53010397
C	12.75084664	16.37711542	3.43317409
H	12.50524281	17.01153721	2.58522952
H	12.47231815	15.35715835	3.18153394
H	13.82503189	16.41099668	3.59389962
C	10.28517876	18.85245593	3.13405198
H	11.33545559	19.05817862	3.26790926
C	9.54739706	19.75316782	2.37093784
C	10.07481704	20.94991866	1.7747665
C	9.01563758	21.58652645	1.18565918
C	7.86417744	20.75713604	1.41663439
C	11.51643644	21.32977449	1.76035726
H	11.95339265	21.18044158	2.75061549
H	11.61266679	22.38515615	1.50288844
C	12.296134	20.50112145	0.7347441
H	13.34560931	20.78268016	0.7446028
H	11.90440627	20.67082084	-0.26454105
H	12.21745989	19.43906719	0.95344391
C	9.0168806	22.82525043	0.35862439
H	9.87096369	23.44572225	0.63201806
H	8.10744106	23.39728515	0.54784101
C	9.10114969	22.48849113	-1.13335954
H	8.26456067	21.86132793	-1.43245627
H	10.02189822	21.9542754	-1.35064122

H	9.08431791	23.39924743	-1.72598632
C	6.58010979	21.0357878	0.97627312
H	6.40279587	21.98404148	0.49349475
C	5.52926552	20.14842146	1.07311711
C	4.22363732	20.30042815	0.51088025
C	3.56285451	19.11245104	0.73876341
C	4.47356162	18.2727549	1.45925453
C	3.7498576	21.50839003	-0.22314588
H	2.66396312	21.59551682	-0.14035861
H	4.18441948	22.4024321	0.22964693
C	4.13643419	21.44764168	-1.70348845
H	3.66771614	20.59334719	-2.18512563
H	5.21380679	21.3526776	-1.81296161
H	3.81273754	22.35090363	-2.21379196
C	2.24568442	18.74203658	0.13529357
H	1.54846681	18.3400638	0.87315784
H	1.79367335	19.64306971	-0.27982516
C	2.44377712	17.72145313	-0.98680206
H	2.84859472	16.79154475	-0.59308138
H	3.14230754	18.10846386	-1.72415046
H	1.50029023	17.51260004	-1.48514662
C	4.2868656	16.89780082	1.878059
C	5.41062107	16.04722983	1.90234983
H	6.37839356	16.45224535	1.59306696
C	5.29948476	14.70754958	2.20388736
H	6.16495944	14.06498645	2.18703342
C	4.04776845	14.19467441	2.52351968
C	3.57132729	12.9023504	2.91058741
C	4.19816553	11.68847136	3.1625386
H	5.26621077	11.59077635	3.05027804
C	3.43142029	10.62184794	3.5679187
H	3.92007682	9.70176631	3.83461869
C	2.03090141	10.71423909	3.68709642
C	1.3972784	11.9372208	3.46897221
H	0.3214632	11.9985022	3.53340278
C	2.17344831	13.01936853	3.09913753
C	2.91581492	15.04735187	2.51469631
C	3.02916023	16.38833671	2.20700876
H	2.16013143	17.02444287	2.23801603
H	-1.55881321	12.75682792	4.22276003
Co	6.45927489	20.85045397	5.69543024
Co	7.0626915	18.31010841	2.7484465
O	6.23450328	19.45517655	4.22196676
N	7.26982698	22.31609519	4.71824609

N	8.0914191	20.19260819	6.34317942
N	5.46228081	19.68173353	6.80046588
N	-0.02834829	22.04568471	5.57651989
H	-0.7315296	21.32894518	5.63438774
N	-3.69657787	25.5918827	3.72549256
N	-5.54843038	24.26685676	2.10084466
N	-3.63045278	22.39720591	1.64421947
N	2.25701548	21.36209274	2.86937502
H	2.45156023	20.3769716	2.81275638
C	6.6887078	23.28219268	3.98440427
C	7.66597114	24.2105537	3.48090721
C	8.8830381	23.76919589	3.93119951
C	8.62266892	22.58317303	4.7003954
C	7.44908535	25.38502295	2.57983988
H	6.48928426	25.29314566	2.06893642
H	8.23018505	25.38450159	1.81592346
C	7.5248381	26.72389463	3.32103267
H	7.26215219	27.53655443	2.64851062
H	8.53195455	26.89805936	3.68755813
H	6.85143584	26.75074334	4.1751213
C	10.223222	24.38870581	3.7486135
H	10.19174935	25.08894452	2.91321513
H	10.95783709	23.61576215	3.51581827
C	10.6686525	25.13687244	5.0104226
H	11.64319404	25.59007431	4.85001513
H	10.7344083	24.46033819	5.85883402
H	9.96063941	25.92241549	5.26113094
C	9.5812111	21.81640753	5.31040829
H	10.61172063	22.10545139	5.17687627
C	9.32758294	20.67992499	6.07330554
C	10.33707149	19.84818748	6.66910049
C	9.67761005	18.80267042	7.25746171
C	8.27609602	19.02549001	7.02672943
C	11.79541466	20.15771282	6.68391578
H	12.35623826	19.25850575	6.94137991
H	12.11870471	20.48790301	5.69381223
C	12.11850652	21.24840887	7.70982324
H	11.57051153	22.16154453	7.49105179
H	13.18244507	21.46912755	7.70042472
H	11.84461438	20.92047042	8.70895427
C	10.23617845	17.69626141	8.08348175
H	11.27785079	17.5261841	7.80928419
H	9.68091315	16.776538	7.89400746
C	10.16107479	18.03397695	9.57573344

H	9.13206319	18.21799576	9.87563972
H	10.74361295	18.9248901	9.79320716
H	10.55579657	17.2124502	10.16755706
C	7.2546711	18.19907809	7.46729833
H	7.52289919	17.27228389	7.94980191
C	5.91700812	18.51928487	7.3713131
C	4.81948638	17.79652866	7.93429445
C	3.69497653	18.56053914	7.7070687
C	4.13046607	19.71998093	6.98620503
C	4.93991263	16.50463608	8.66842681
H	5.72965688	15.90130273	8.21513181
H	4.00904611	15.93860984	8.58650201
C	5.25908505	16.73307644	10.14845683
H	4.45684775	17.28582584	10.63051493
H	6.1789782	17.30205695	10.25705085
H	5.37616631	15.78084959	10.65897058
C	2.35219157	18.29905566	8.31116709
H	2.35440547	17.29155672	8.72763051
H	1.54852679	18.34295588	7.57333993
C	2.06961448	19.3009177	9.43203079
H	2.0119731	20.31299401	9.03706776
H	2.86779532	19.27101179	10.16920269
H	1.13334152	19.06293121	9.9309263
C	3.34517494	20.86386507	6.56721089
C	3.96637314	22.12890132	6.54148046
H	5.0130847	22.20263185	6.85015818
C	3.26451536	23.27523053	6.23922844
H	3.74861593	24.23836059	6.25491006
C	1.91571639	23.17015295	5.92031734
C	0.90885346	24.10978282	5.53267198
C	0.92272409	25.47562453	5.27928972
H	1.83278949	26.04330765	5.39056332
C	-0.24198168	26.08308526	4.87384445
H	-0.21949352	27.12439785	4.60617026
C	-1.45136812	25.37058957	4.75593995
C	-1.46723562	23.99353834	4.97547123
H	-2.40060161	23.4549477	4.91210235
C	-0.28716892	23.37643822	5.34544929
C	1.28810299	21.89954392	5.93077165
C	1.99250308	20.7530556	6.23919722
H	1.50228574	19.79409943	6.209479
C	-2.68042393	26.08123831	4.45863127
C	-2.98825082	27.3935487	4.96205598
C	-4.24839672	27.69169713	4.51264835

C	-4.68147276	26.557015	3.743858
C	-2.16203675	28.25694116	5.86218012
H	-2.8147937	28.68511283	6.62655019
H	-1.40938689	27.65387218	6.3725816
C	-1.4917326	29.41775771	5.11997258
H	-0.91464458	29.070523	4.26553828
H	-0.82580854	29.9535014	5.79170625
H	-2.23867352	30.11557748	4.75379373
C	-5.02941108	28.94458261	4.69571411
H	-6.06733276	28.70090112	4.92946216
H	-4.61844043	29.51306142	5.53066951
C	-4.99289222	29.81435371	3.43377954
H	-5.4195422	29.28452193	2.58579953
H	-3.97032648	30.08314266	3.18213393
H	-5.55932195	30.7276696	3.59460747
C	-5.90375983	26.44137604	3.13435449
H	-6.60705807	27.24806737	3.26830524
C	-6.31492475	25.35214195	2.37116558
C	-7.61506907	25.21058314	1.77501472
C	-7.63681269	23.9750546	1.18580502
C	-6.34281096	23.39252419	1.41672575
C	-8.66484296	26.26913966	1.76070987
H	-8.7539805	26.72213972	2.75100678
H	-9.62695317	25.82481686	1.50321498
C	-8.33706457	27.35879066	0.73518567
H	-9.10563447	28.12688667	0.74512246
H	-8.28818344	26.93478344	-0.26413793
H	-7.3779566	27.82166242	0.95390956
C	-8.71022477	23.35684333	0.35874452
H	-9.67460253	23.78625185	0.63218964
H	-8.75090872	22.28321474	0.54788229
C	-8.46074781	23.59831171	-1.13322651
H	-7.49932585	23.18739767	-1.43237729
H	-8.45847251	24.66282768	-1.35042756
H	-9.24108906	23.12841074	-1.72587031
C	-5.94212027	22.14118769	0.97628377
H	-6.67467574	21.51356321	0.49342737
C	-4.64822395	21.67479151	1.07308364
C	-4.12707342	20.46810058	0.51078007
C	-2.76786788	20.48978062	0.73869056
C	-2.4960048	21.69828923	1.45923319
C	-4.93632947	19.45385142	-0.22327304
H	-4.46885371	18.46986459	-0.14051528
H	-5.92787834	19.38317802	0.22951284

C	-5.07698064	19.8190747	-1.70360093
H	-4.10277485	19.84030553	-2.18522701
H	-5.53341554	20.7995975	-1.81304259
H	-5.69738295	19.08713713	-2.21393244
C	-1.78850433	19.53425442	0.13525241
H	-1.09182315	19.13139171	0.87314157
H	-2.34283055	18.69231493	-0.27991066
C	-1.00363359	20.21611506	-0.98678794
H	-0.40073224	21.03164288	-0.59302654
H	-1.68800992	20.62756397	-1.72417832
H	-0.35099269	19.50345467	-1.48509135
C	-1.21190654	22.22402346	1.87810049
C	-1.03714113	23.62250429	1.902408
H	-1.87177855	24.25813374	1.59314077
C	0.17864211	24.1960693	2.20392406
H	0.30240223	25.26687149	2.18707806
C	1.24865514	23.36846315	2.52351544
C	2.60607883	23.60198857	2.91053625
C	3.34393979	24.75177692	3.16243867
H	2.89453654	25.72558567	3.05018244
C	4.6510547	24.62104714	3.56774665
H	5.20358519	25.50426972	3.83438248
C	5.27127883	23.36195441	3.68691172
C	4.52893034	22.201737	3.46884156
H	5.01375368	21.2394036	3.53327929
C	3.20366159	22.33286956	3.09906337
C	1.07616234	21.96182775	2.51469614
C	-0.14185949	21.38952256	2.20703277
H	-0.25824655	20.31886813	2.23800923
H	5.29734334	19.2320161	4.22255465
Co	-5.72128738	22.12902253	5.69560207
Co	-3.82286639	23.92171319	2.74866241
O	-4.40027173	22.63218359	4.22253923
N	-7.39575872	22.09819328	4.71824645
N	-5.96758958	23.87139413	6.34334699
N	-4.21048624	21.84994438	6.80042655
N	-3.51245353	15.91296388	5.57641241
H	-2.54015186	15.66235105	5.63430664
N	-4.74929899	10.96317838	3.72526947
N	-2.67566448	10.02204778	2.10080441
N	-2.01557317	12.61791982	1.64423792
N	-4.06310386	18.23408713	2.8692894
H	-3.30724257	18.89514089	2.81272279
C	-7.94184569	21.11190991	3.98434742

C	-9.23447482	21.49407319	3.48090176
C	-9.46079423	22.76873894	3.93126569
C	-8.30348014	23.13624489	4.70045736
C	-10.14315362	20.7190445	2.5798124
H	-9.58367732	19.93380922	2.06885905
H	-10.53326017	21.39580231	1.8159414
C	-11.34051163	20.11515733	3.32098511
H	-11.91295537	19.48137542	2.64842697
H	-11.99490158	20.90023644	3.68756753
H	-11.02704871	19.51849611	4.17503209
C	-10.66740895	23.61961264	3.74875185
H	-11.25810073	23.2422714	2.913342
H	-10.36533991	24.64229416	3.51600369
C	-11.53803672	23.63121181	5.01057565
H	-12.41780008	24.2485877	4.85021039
H	-10.98501309	24.02638756	5.85900131
H	-11.86431795	22.62526751	5.26123745
C	-8.11872714	24.34971912	5.31055037
H	-8.884319	25.0976352	5.17707875
C	-7.00769937	24.69828919	6.07347489
C	-6.79216558	25.98835725	6.66936699
C	-5.5569829	25.93999705	7.25772048
C	-5.04916328	24.61486999	7.0269057
C	-7.78941817	27.09653024	6.68424803
H	-7.29110944	28.03181062	6.94177656
H	-8.23700756	27.21146831	5.6941468
C	-8.8955353	26.83090168	7.71013101
H	-9.41231334	25.89975818	7.49129649
H	-9.61866862	27.64192652	7.70077592
H	-8.4745902	26.75762047	8.70925986
C	-4.87811673	26.97688059	8.08382929
H	-5.25169274	27.96404541	7.80971988
H	-3.80397875	26.95591621	7.89435692
C	-5.13303484	26.74283315	9.57605816
H	-4.77786011	25.7596576	9.87587253
H	-6.19586042	26.80181029	9.7935336
H	-4.61896112	27.4953982	10.16795393
C	-3.82273527	24.14349911	7.46742922
H	-3.15421403	24.83918608	7.94992341
C	-3.43116817	22.82496216	7.37134206
C	-2.25645511	22.23585602	7.93425879
C	-2.35584264	20.88000167	7.70699203
C	-3.57769983	20.67742896	6.98613813
C	-1.19787099	22.98608225	8.66842278

H	-1.07027512	23.97171286	8.21517536
H	-0.24223262	22.46297156	8.58645557
C	-1.55526313	23.14819775	10.14846947
H	-1.63279248	22.17704132	10.63048589
H	-2.50797907	23.6603159	10.25710746
H	-0.78916429	23.72571239	10.65899703
C	-1.45799736	19.84786103	8.31109358
H	-0.58656905	20.35352677	8.72752551
H	-1.09420259	19.12990881	7.57326822
C	-2.18431835	19.10222119	9.43198331
H	-3.03197496	18.54623917	9.03704055
H	-2.55751912	19.80843608	10.16913322
H	-1.51006458	18.41040752	9.93089997
C	-4.17571139	19.42541214	6.56715994
C	-5.58186809	19.33088078	6.54140108
H	-6.1690743	20.20048658	6.85008624
C	-6.22369753	18.14990556	6.23910264
H	-7.29984392	18.08759472	6.25475872
C	-5.45830358	17.03435184	5.92017419
C	-5.76861846	15.69258327	5.53248223
C	-6.95840405	15.02169428	5.27903055
H	-7.90506254	15.52600168	5.39028006
C	-6.90212756	13.70931463	4.87353353
H	-7.81516989	13.20816455	4.6057888
C	-5.68039877	13.0181903	4.75566135
C	-4.4799078	13.69295627	4.97527526
H	-3.54679827	13.15392086	4.91195248
C	-4.53551273	15.02346166	5.34528578
C	-4.04411599	17.12610974	5.93067757
C	-3.40342148	18.30936413	6.23914638
H	-2.32783369	18.36429022	6.20943144
C	-5.68128548	11.59848513	4.4582969
C	-6.66393359	10.6757117	4.96152591
C	-6.29197742	9.43533785	4.51213099
C	-5.09266102	9.62766466	3.743536
C	-7.82491889	10.95947219	5.86146364
H	-7.86948612	10.18001457	6.62575553
H	-7.67905421	11.91277167	6.37197884
C	-9.16523195	10.95963936	5.11901045
H	-9.15290375	11.6331103	4.26464133
H	-9.96228514	11.26841007	5.79062747
H	-9.39602273	9.96389402	4.75269447
C	-6.98650688	8.13249833	4.69503495
H	-6.25653504	7.35548008	4.92888161

H	-7.68445138	8.20412942	5.52987453
C	-7.75778764	7.72925964	3.43295721
H	-7.08547134	7.62473562	2.58508458
H	-8.50182984	8.48041873	3.18121368
H	-8.26552585	6.78203835	3.59366779
C	-4.3812455	8.62698207	3.13412198
H	-4.72819042	7.614548	3.26801259
C	-3.23226008	8.81557374	2.37109545
C	-2.45945948	7.76043292	1.77505553
C	-1.37854704	8.35941688	1.18597231
C	-1.52117646	9.77131573	1.41683605
C	-2.85122633	6.32200359	1.76072816
H	-3.19908601	6.01831439	2.75098486
H	-1.9853015	5.71100285	1.50337031
C	-3.95862099	6.06094867	0.73505545
H	-4.23946572	5.01128273	0.74497985
H	-3.61573544	6.31528105	-0.2642261
H	-4.83909927	6.66007852	0.95364156
C	-0.30630643	7.73896995	0.35906786
H	-0.19597237	6.68910388	0.63255506
H	0.64376932	8.24061192	0.54832515
C	-0.63996372	7.83423255	-1.13295125
H	-0.76483725	8.87229033	-1.43214387
H	-1.56293675	7.30388317	-1.35026589
H	0.15726214	7.393424	-1.72547602
C	-0.63786358	10.74403411	0.97642213
H	0.27202299	10.42347919	0.49367
C	-0.88100494	12.09776651	1.07314987
C	-0.09661126	13.15247207	0.51083022
C	-0.79509268	14.31869518	0.73864948
C	-1.97761187	13.94983191	1.45918022
C	1.1864145	12.95881695	-0.22318159
H	1.8047747	13.85569842	-0.14043745
H	1.74343294	12.13549966	0.22965069
C	0.94051656	12.65432415	-1.70350636
H	0.43499067	13.48734458	-2.18518199
H	0.31963416	11.76873604	-1.81293297
H	1.88461999	12.48304574	-2.21380286
C	-0.45736916	15.64459694	0.1351234
H	-0.45686188	16.4494128	0.87296543
H	0.54893183	15.58554332	-0.28004852
C	-1.44034419	15.98327322	-0.98692387
H	-2.44806535	16.09759985	-0.59315847
H	-1.45444144	15.18482204	-1.72427194

H	-1.14954268	16.90479803	-1.4852805
C	-3.07501264	14.79898775	1.87798593
C	-4.37350513	14.25107439	1.90223556
H	-4.50662327	13.21044641	1.59295677
C	-5.47814215	15.01716933	2.2037222
H	-6.46735712	14.58893366	2.18683665
C	-5.29645217	16.35762581	2.52334957
C	-6.17743174	17.41640144	2.91037354
C	-7.54211441	17.48049115	3.16224962
H	-8.16074359	16.60438982	3.04994827
C	-8.08247925	18.67782983	3.56759371
H	-9.12363963	18.71469867	3.8342237
C	-7.30220109	19.84450977	3.68681564
C	-5.92624805	19.78174537	3.46875926
H	-5.3352753	20.68278602	3.53320004
C	-5.37715464	18.56848635	3.09895497
C	-3.9920314	16.91158148	2.51458507
C	-2.88736798	16.14292179	2.20694513
H	-1.90196523	16.57746094	2.23797747
H	-3.73851317	21.93207895	4.22289094
